# Metabolomic alterations in invasive ductal carcinoma of breast: A comprehensive metabolomic study using tissue and serum samples

**DOI:** 10.18632/oncotarget.23626

**Published:** 2017-12-23

**Authors:** Tushar H. More, Sourav RoyChoudhury, Joel Christie, Khushman Taunk, Anupama Mane, Manas K. Santra, Koel Chaudhury, Srikanth Rapole

**Affiliations:** ^1^ Proteomics Lab, National Center for Cell Science, Ganeshkhind, Pune 411007, MH, India; ^2^ Savitribai Phule Pune University, Ganeshkhind, Pune 411007, MH, India; ^3^ School of Medical Science and Technology, Indian Institute of Technology, Kharagpur 721302, WB, India; ^4^ Grant Medical Foundation, Ruby Hall Clinic, Pune 411001, MH, India; ^5^ Cancer Biology and Epigenetics Lab, National Center for Cell Science, Ganeshkhind, Pune 411007, MH, India

**Keywords:** Invasive ductal carcinoma, targeted metabolomics, untargeted metabolomics, tissue, serum

## Abstract

Invasive ductal carcinoma (IDC) is the most common type of breast cancer and the leading cause of breast cancer related mortality. In the present study, metabolomic profiles of 72 tissue samples and 146 serum samples were analysed using targeted liquid chromatography multiple reaction monitoring mass spectrometry (LC-MRM/MS) and untargeted gas chromatography mass spectrometry (GC-MS) approaches. Combination of univariate and multivariate statistical treatment identified significant alterations of 42 and 32 metabolites in tissue and serum samples of IDC, respectively when compared to control. Some of the metabolite changes from tissue were also reflected in serum, indicating a bi-directional interaction of metabolites in IDC. Additionally, 8 tissue metabolites and 9 serum metabolites showed progressive change from control to benign to IDC suggesting their possible role in malignant transformation. We have identified a panel of three metabolites viz. tryptophan, tyrosine, and creatine in tissue and serum, which could be useful in screening of IDC subjects from both control and benign. The metabolomic alterations in IDC showed perturbations in purine and pyrimidine metabolism, amino sugar metabolism, amino acid metabolism, fatty acid biosynthesis etc. Comprehensively, this study provides valuable insights into metabolic adaptations of IDC, which can help to identify diagnostic markers as well as potential therapeutic targets.

## INTRODUCTION

Breast cancer is the most common malignancy observed in woman throughout the world with a prevalence of around 23% [1]. Besides, it is also the leading cause of cancer-related deaths in women with a mortality rate of about 14% [2]. High breast cancer related mortalities in developing countries like India can be attributed to early age cancer incidences, late diagnosis, and inefficiency of the existing therapies [3]. Breast cancer is a heterogeneous disease and can be classified into various subtypes based on the histological and molecular characteristics [4, 5]. Invasive ductal carcinoma (IDC) also referred as infiltrating ductal carcinoma is a type of breast cancer which originates in the ductal epithelium of the breast and invades the surrounding tissues [6]. It is the most prevalent breast cancer type and accounts for about 70% of the total breast cancer cases [7]. If not detected at an early stage, IDC can potentially metastasize to other parts of the body through the lymph node spread or in the more advanced stage through the blood stream [6]. It is notable that early diagnosis significantly increases the success of therapies and ultimately the long-term survival of breast cancer individuals [8]. Current methods used for the diagnosis and surveillance of breast cancer such as mammography and histopathology have some limitations [9, 10]. Moreover, lack of specific diagnostic markers and unavailability of proper screening protocols at healthcare facilities significantly affects early diagnosis [11]. Therefore, it necessitates the discovery of more specific markers for the early detection and new molecular targets for developing therapies for IDC.

Identifying disease-specific molecular signatures through ‘omics’ based platforms have substantially helped in understanding disease pathogenesis [12]. The ‘omics’ based approaches, including genomics, proteomics, transcriptomics have been extensively used to understand tumour biology [13]. Metabolomics, a recent development in ‘omics’ platforms, is rapidly emerging field in the cancer research. The metabolome, which refers to the total set of metabolites of a living system, is the ultimate product of all the biological processes. Hence, identifying metabolomic alterations using metabolomics approach has enormous potential to impact cancer theranostics [14]. Metabolomics and cancer share close connectivity, cancer cell undergoes profound metabolic rearrangements in numerous physiological processes towards tissue remodelling, tumour growth, and cancer metastasis [15–17]. Although metabolomic alterations are the consequence of genomic transformation adapted by cancer cells, strikingly, in some cases it is the foremost cause for cancer [17]. Metabolomics offers unique insights into the regulation of small-molecule metabolites and the signaling pathways underlying various biological processes. Thus, metabolomics has been implemented by researchers in quest of identifying new diagnostic markers and potential therapeutic targets for cancer including leukemia [18], colorectal cancer [19], and hepatocellular carcinoma [20]. Recently, some of the metabolomics studies have also been performed in breast cancer, including study of various breast cancer subtypes [21, 22]. However, none of these studies have focused on identifying metabolomic alterations specific to IDC.

In the present study, we hypothesised that certain metabolic alterations exist in the IDC, which help in the progression of IDC. The metabolic demand of the proliferating cancer cell is fulfilled by either their synthesis or their uptake from the surrounding micro-environment. As tumour tissues are the hub of metabolic turnover and blood represents the pool of metabolites from various tissues, complementing tissue metabolomic profile with serum could provide a composite metabolomic snapshot for IDC [23]. In past, metabolomic studies were conducted either on serum, plasma or tissue samples, however, none of them reported overall metabolic changes in serum and tissue from the same subjects and tried to interconnect them in a single study [21, 22, 24]. Hence, in the present study, we intended to perform a comprehensive metabolic profiling of both tissue and serum samples in an anticipation of getting a holistic view of IDC metabolism. Additionally, the altered serum metabolites identified in this study can have a potential to be used as a less invasive alternative as a diagnostic and prognostic markers for IDC. Another important topic of continuous debate in breast cancer is related to benign to malignant transformation of breast tumour. Although this phenomenon is very uncommon but there are some reports regarding the pathways involved in transformation of a benign into malignant tumour [25–27]. A well designed metabolomics study can offer unique insights into the regulation of small-molecule metabolites in IDC and the signalling pathways underlying. The metabolic alterations in benign subjects were also examined in anticipation of identifying the metabolic alterations which may have a possible role in benign to malignant transition. Mass spectrometry (MS) based metabolomics approaches can be broadly classified into two main categories viz. targeted and untargeted metabolomics [28]. Targeted approach is a quantitative measurement of known set of metabolites related to a specific metabolic class or pathway, thus, a prior knowledge about metabolites including fragmentation pattern is essential. In contrast, untargeted approach refers to the measurement of all possible metabolites detected in a given sample, including the unknowns. Gas chromatography mass spectrometry (GC-MS) is one of the most suitable methods of choice for untargeted analysis and liquid chromatography multiple reaction monitoring mass spectrometry (LC-MRM/MS) is widely used for targeted analysis [29]. It is evident that the parallel use of untargeted GC-MS and targeted LC-/MRM-MS analysis is the best choice in profiling of compounds from different classes [21].

In this study, metabolomic profiling of 24 tissue samples each of malignant IDC, benign neoplasm and normal tissue samples were studied using targeted LC-MRM/MS and untargeted GC-MS approaches. Further, serum metabolomic profiles of 76 IDC, 33 benign and 33 age and gender-matched healthy controls were also acquired to correlate the findings from tissue analysis. Here, we have shown that existence of distinct metabolomic profile that marked for IDC, differentiating them from healthy controls and benign subjects. Moreover, some of the metabolite changes from tissue were also reflected in serum, emphasizing bi-directional interaction of blood with tumour tissue.

## RESULTS

### Clinical characteristic of study population

**Figure 1:**
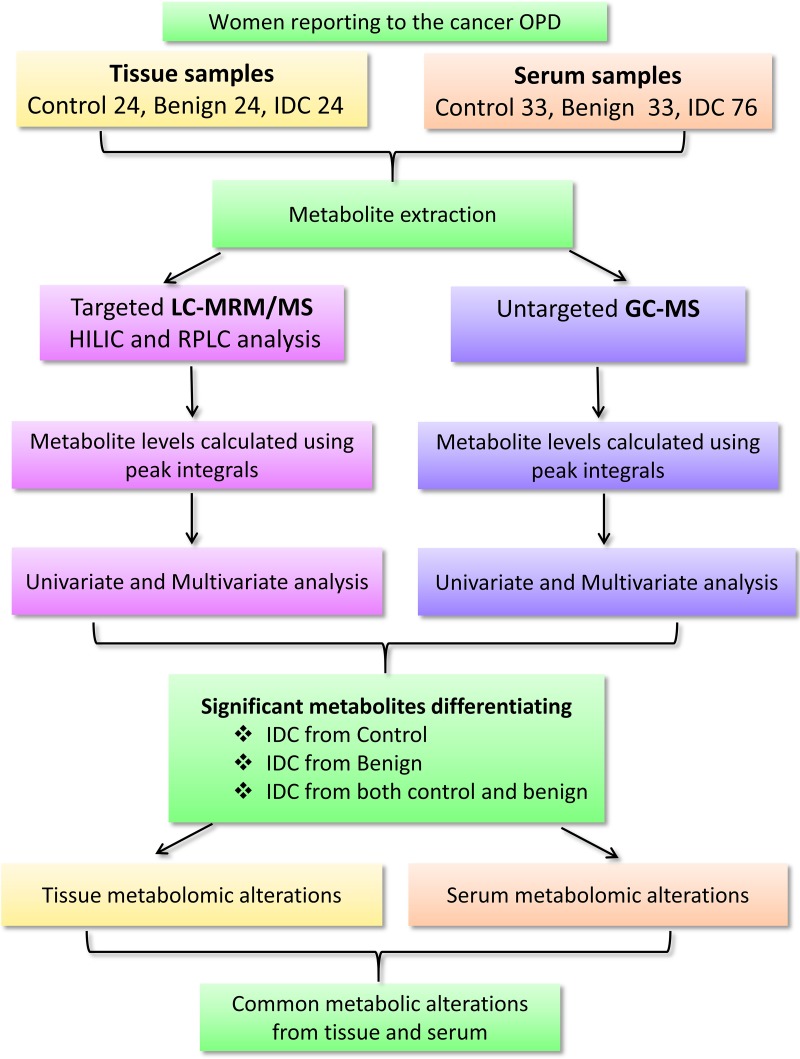
A flowchart depicting the outline of the study Workflow and steps evolved for the metabolomic study conducted on tissue (24 IDC, 24 benign and 24 normal) and serum (76 IDC, 33 benign and 33 control) samples using targeted liquid chromatography multiple reaction monitoring mass spectrometry (LC-MRM/MS) and untargeted gas chromatography mass spectrometry (GC-MS) approaches are shown. [Legends: HILIC-Hydrophilic interaction chromatography (HILIC column), RPLC T3: Reverse phase chromatography (T3 column)].

For tissue metabolomics analysis, a study cohort comprising 24 IDC, 24 benign and 24 controls (normal tissue) were examined. To elucidate the metabolomic alterations in serum, a total of 142 subjects including 76 IDC, 33 benign and 33 healthy subjects were recruited (Figure 1). IDC subjects were having invasive ductal lesion in breast, which was further confirmed by histopathology examination after the breast biopsy. Benign subjects were having non-malignant lesions such as fibro adenoma, chronic inflammation, granuloma and any other type of lesions in breast. The subjects enrolled for the study were freshly diagnosed for IDC who did not undergo any therapeutic intervention such as neoadjuvant chemotherapy or radiotherapy. The tumours were appropriately stage and/or size matched according to new TNM (tumour, node and metastasis) staging criteria for breast cancer [30]. Control tissue used in this study was the normal tissue adjacent to the tumour (2-5 cm away), obtained from same patient whereas control sera were collected from healthy women with no apparent metabolic disease such as diabetes and hypertension. Both tissue and serum samples were collected from the same patients. Diagnosis of the all the patients was confirmed by histopathology examination. The overall demographic and histological characteristics of the subjects enrolled for this study are mentioned in Table 1.

**Table 1:** Demographics and histological features of samples used for this metabolomics study

Description	TISSUE	SERUM
**Healthy Controls**		
No. of cases	24	33
Age (average ± standard deviation)	50 ± 8	48 ± 10
**Benign Samples**		
No. of cases	24	33
Age (average ± standard deviation)	47 ± 10	45 ± 13
Subtype		
Fibro adenoma	11	16
Chronic inflammation	5	7
Granuloma	3	4
Other	5	6
**Breast cancer samples**		
No. of cases	24	76
Age (average ± standard deviation)	54 ± 10	53 ± 12
*Type*		
Invasive ductal carcinoma	24	76
*Tumour grade*		
Grade 1	15	26
Grade 2	9	50
*Tumour stage*		
Stage II (T2N1M0, T3N0M0)	17	52
Stage III (T0N2M0, T1N2M0, T2N2M0, T3N1M0, T3N2M0)	7	24
*Subtype*		
LUMINAL A	8	25
LUMINAL B	9	23
HER2 ENRICHED	4	13
TRIPLE NEGATIVE	3	15

### Tissue metabolomic profiling

Initially, we performed a metabolomic profiling of tissue samples (n = 72) from IDC, benign and peripheral normal tissues, in order to identify the IDC specific metabolic alterations in tissue. The results obtained from tissue metabolomic profiling are described as follows.

#### Targeted LC-MRM/MS

**Figure 2:**
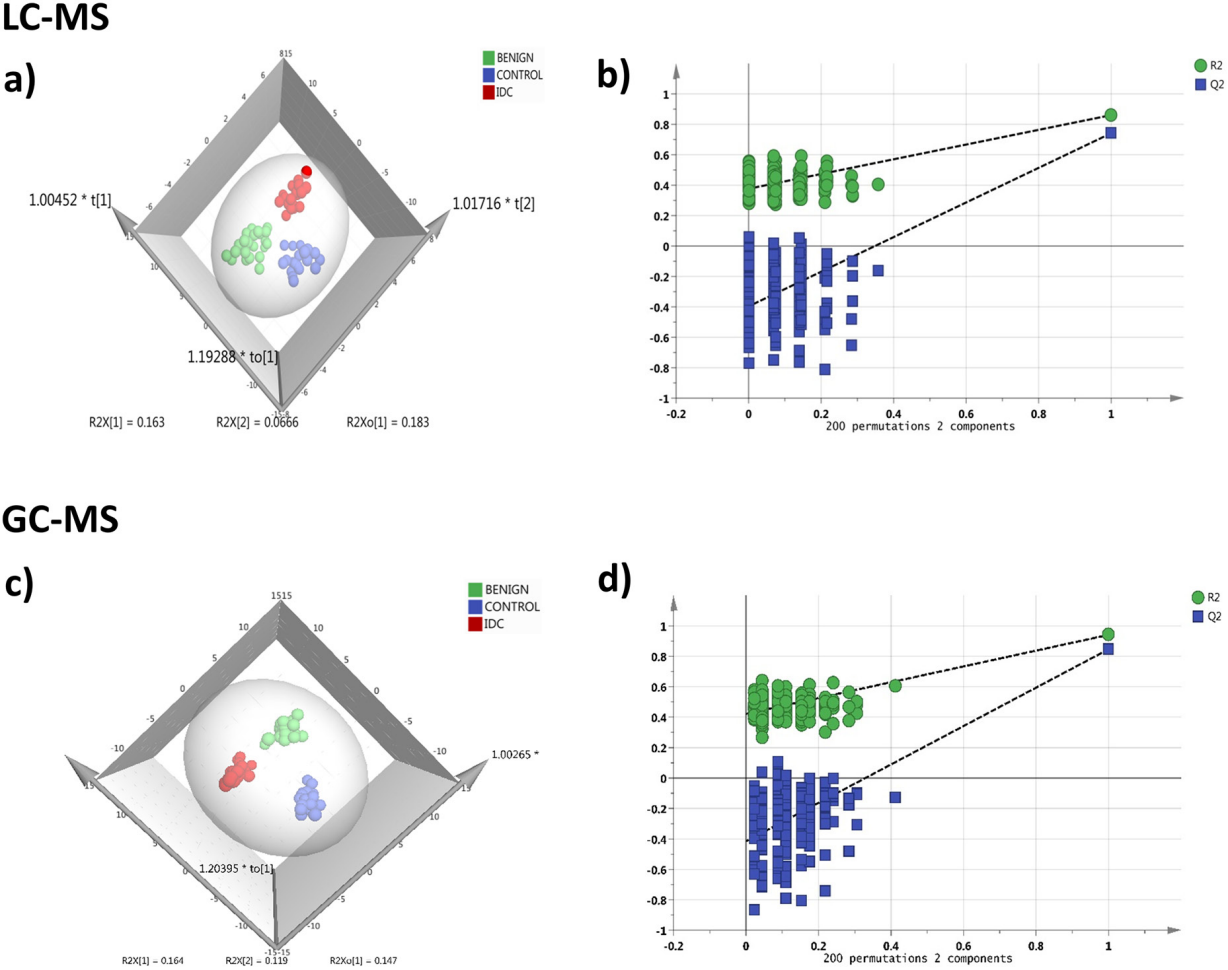
Tissue metabolomics multivariate analysis LC-MRM/MS: **(a)** OPLS-DA score plot of tissue LC-MRM/MS data from IDC subjects (n = 24, red), benign breast patients (n = 24, green) and healthy controls (n = 24, blue), **(b)** plot obtained after performing a random permutation test with 200 permutations on OPLS-DA model (R2Y = 0.86, Q2 = 0.77), R2 is the explained variance, and Q2 is the predictive ability of the model. Low value of R2Y and Q2-intercepts at 0.36 and -0.45 depicts the high predictability of the model, GC-MS: **(c)** OPLS-DA score plot of tissue GC-MS data for IDC subjects (n = 24, red), benign breast patients (n = 24, green) and healthy controls (n = 24, blue), **(d)** plot obtained after performing a random permutation test with 200 permutations on OPLS-DA model (R2Y = 0.94, Q2 = 0.88).

In LC-MRM/MS analysis, out of the 108 metabolites targeted, 91 metabolites were detected consistently at a sufficient level for quantitation with minimum 30% base peak intensity. The representative chromatograms obtained from targeted LC-MRM/MS are shown in Supplementary Figure 1. The peak area data matrix was pre-processed using sample median normalization, cube root transformation, and range scaling methods. Multivariate analysis (MVA) was performed on the normalized dataset. Clear discrimination was observed within the three group's viz. IDC, benign and control (normal tissue) in orthogonal partial least squares discriminant analysis (OPLS-DA) model (Figure 2a). The cumulative R2 and Q2 values of the model were 0.86 and 0.77 respectively indicating a good fitting and high predictive ability of the model. The obtained OPLS-DA model was further cross-validated using permutation analysis. The R2 and Q2 values of the originally obtained model were better than the 200 randomly permutated models indicating good predictive capacity of the obtained OPLS-DA model (Figure 2b). Hierarchical clustering analysis (HCA) showed distinct clusters for IDC, benign and controls based on the differences between metabolite concentrations in these groups (Supplementary Figure 3a).

#### Untargeted GC-MS analysis

During GC-MS analysis, 218 metabolites were detected out of which 148 had an occurrence frequency of at least 80% among the samples and were considered for further data analysis. A representative GC-MS chromatogram is shown in Supplementary Figure 2. The peak area data matrix was sum normalized, cubic root transformed and then auto-scaled. OPLS-DA plot (R2 0.94 and Q2 0.88) showed clear discrimination between IDC, benign and control groups (Figure 2c). Cross-validation analysis using 200 random permutations is shown in Figure 2d which confirms the good predictive ability of the generated OPLS-DA model. HCA plot depicts a distinct clusters between IDC, benign and healthy controls (Supplementary Figure 3b).

### Identification of significantly altered metabolites in tissue IDC against control

**Figure 3:**
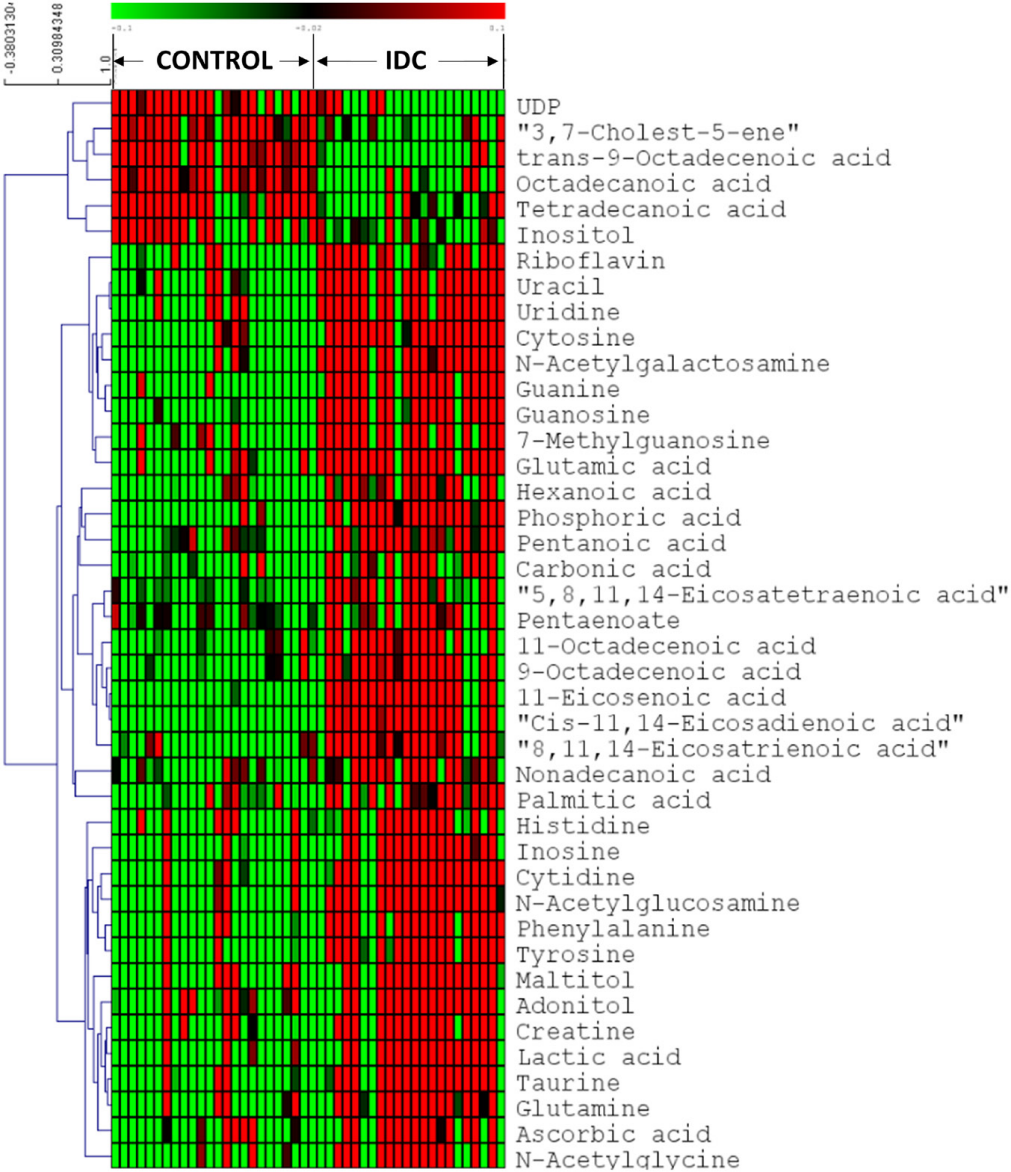
Heatmap of 42 differential metabolites between IDC and controls in tissue samples The colours from green to red indicate the increased amount of metabolites (Control: Normal tissue, IDC: Invasive ductal carcinoma).

**Figure 4:**
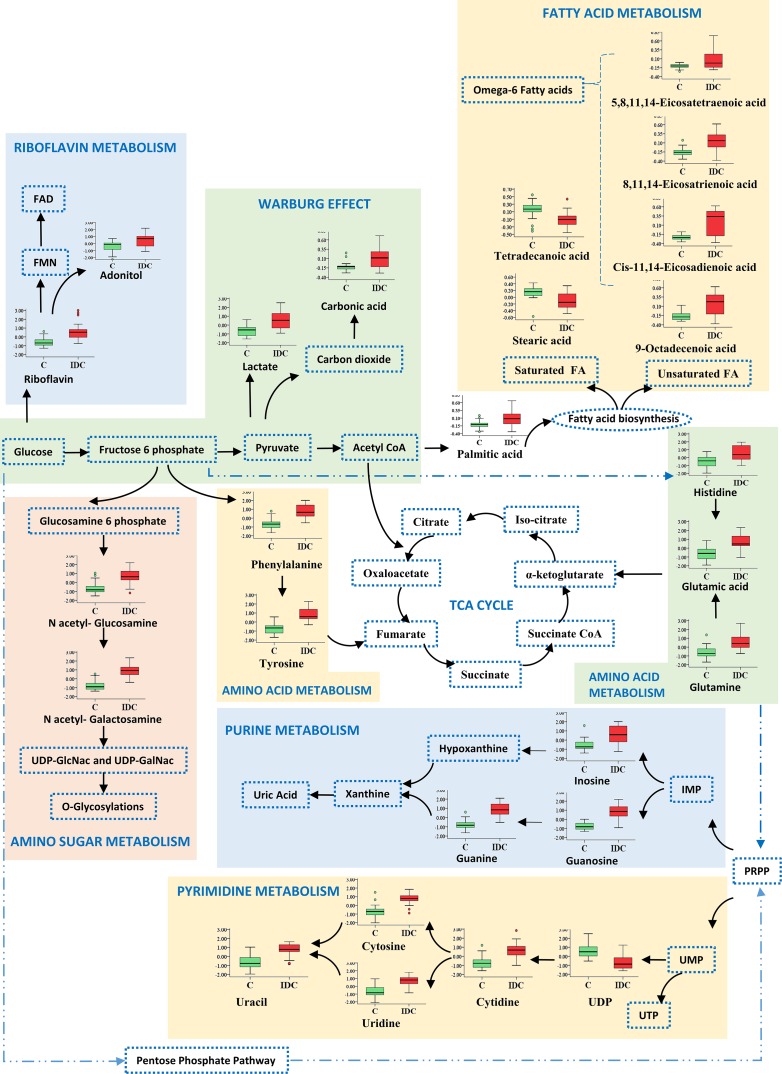
Metabolic pathway network map of significantly altered metabolites from IDC against control analysis in tissue samples Metabolites identified in this study are shown along with a box plot illustrating normalized concentration differences of metabolite in IDC tissue (red box) and control (green box). While, metabolites not identified in this study are represented with a dotted blue lines.

First, we screened for the metabolomic alterations that could discriminates IDC from controls. MVA plots of IDC against control obtained from LC-MRM/MS and GC-MS are shown in Supplementary Figure 4. The differentially expressed metabolites were identified using the combination of univariate and multivariate statistics. Metabolites with VIP score ≥ 1.2 from OPLS-DA model, were considered as important features for class separation. Further, metabolites with p < 0.05 were selected upon student's *t*-test analysis and the p-values were then adjusted for multiple hypotheses testing using FDR calculations. The collective results from VIP and *t*-test revealed significant alterations of 42 metabolites, including 24 metabolites from LC-MRM/MS and 18 metabolites from GC-MS in IDC tissue as compared to control samples (Table 2). Amongst the 42 significant metabolites, 36 were up-regulated and 6 were down-regulated (Table 2). These include amino acids, nucleotides, fatty acids, amino sugar derivatives, organic acids etc. Heat map of these 42 differentially altered metabolites between IDC tissue and control is shown in Figure 3. The metabolic pathway network map of the significantly altered metabolites from IDC against control tissue analysis is presented in Figure 4.

**Table 2:** Tissue metabolites differentiating IDC from control subjects a) Differential metabolites identified using LC-MRM/MS, b) Differential metabolites identified using GC-MS

S. No	Metabolite	HMDB ID	VIP	p-value	FDR	FC	AUC
**a) LC-MS**							
1	Guanine	HMDB00132	1.72	5.16E-11	1.57E-09	6.37	0.97
2	N-Acetylgalactosamine	HMDB00212	1.69	7.65E-12	6.96E-10	4.87	0.97
3	Guanosine	HMDB00133	1.68	2.16E-11	9.81E-10	7.14	0.96
4	UDP	HMDB00295	1.59	9.13E-07	5.93E-06	0.59	0.88
5	Uracil	HMDB00300	1.57	3.45E-08	4.48E-07	2.71	0.91
6	Uridine	HMDB00296	1.57	4.52E-08	5.14E-07	2.63	0.92
7	Cytosine	HMDB00630	1.55	2.51E-08	4.48E-07	3.97	0.91
8	Tyrosine	HMDB00158	1.54	1.25E-09	2.84E-08	8.73	0.96
9	Taurine	HMDB00251	1.52	5.23E-07	3.66E-06	5.62	0.88
10	Phenylalanine	HMDB00159	1.49	5.48E-08	5.54E-07	3.98	0.92
11	Creatine	HMDB00064	1.48	1.26E-05	6.36E-05	4.32	0.85
12	N-Acetylglucosamine	HMDB00215	1.48	4.00E-07	3.03E-06	4.82	0.88
13	Glutamine	HMDB00641	1.48	7.01E-06	3.75E-05	4.60	0.87
14	7-Methylguanosine	HMDB01107	1.48	2.97E-08	4.48E-07	4.54	0.92
15	Lactic acid	HMDB00190	1.46	4.58E-06	2.61E-05	4.35	0.85
16	Glutamic acid	HMDB00148	1.46	1.89E-07	1.72E-06	2.39	0.91
17	Cytidine	HMDB00089	1.44	3.74E-07	3.03E-06	5.16	0.89
18	Riboflavin	HMDB00244	1.33	3.94E-06	2.39E-05	3.79	0.90
19	Adonitol	HMDB00508	1.32	6.12E-04	2.23E-03	2.22	0.78
20	Inosine	HMDB00195	1.32	3.04E-05	1.46E-04	4.95	0.84
21	Maltitol	HMDB02928	1.31	1.71E-03	4.85E-03	2.12	0.78
22	Histidine	HMDB00177	1.30	1.59E-04	6.89E-04	3.28	0.79
23	N-Acetylglycine	HMDB00532	1.28	4.76E-04	1.80E-03	3.09	0.77
24	Ascorbic acid	HMDB00044	1.25	2.13E-03	5.69E-03	3.07	0.76
**b) GC-MS**							
25	Phosphoric acid	HMDB02142	1.71	7.17E-08	7.89E-06	10.72	0.83
26	Cis-11,14-Eicosadienoic acid	HMDB05060	1.69	9.46E-07	3.47E-05	30.41	0.70
27	11-Eicosenoic acid	HMDB34296	1.67	2.18E-06	5.98E-05	51.94	0.75
28	9-Octadecenoic acid	HMDB00207	1.53	5.53E-05	6.90E-04	7.14	0.74
29	8,11,14-Eicosatrienoic acid	HMDB02925	1.53	1.35E-05	2.48E-04	6.87	0.70
30	trans-9-Octadecenoic acid	HMDB00573	1.51	4.11E-06	9.04E-05	0.28	0.89
31	Pentanoic acid	HMDB00892	1.42	2.64E-05	4.15E-04	11.99	0.78
32	Nonadecanoic acid	HMDB00772	1.41	1.07E-04	1.07E-03	4.83	0.75
33	11-Octadecenoic acid	HMDB03231	1.40	4.03E-04	2.63E-03	7.07	0.82
34	3,7-Cholest-5-ene	HMDB00941	1.34	3.49E-04	2.63E-03	0.48	0.81
35	Hexanoic acid	HMDB00535	1.32	2.78E-04	2.35E-03	11.87	0.69
36	Octadecanoic acid	HMDB00827	1.30	5.65E-05	6.90E-04	0.39	0.68
37	5,8,11,14-Eicosatetraenoic acid	HMDB01043	1.29	1.12E-03	5.34E-03	8.63	0.65
38	Inositol	HMDB00211	1.26	4.30E-04	2.63E-03	0.22	0.84
39	Tetradecanoic acid	HMDB00806	1.24	1.71E-04	1.57E-03	0.34	0.81
40	Carbonic acid	HMDB03538	1.23	8.48E-04	4.44E-03	7.98	0.76
41	Palmitic acid	HMDB00220	1.23	1.83E-03	6.71E-03	3.78	0.67
42	Eicosapentaenoic acid	HMDB01999	1.20	5.92E-03	1.59E-02	4.55	0.70

HMDB ID: Metabolite ID obtained from HMDB database, VIP score: variable of importance score obtained from OPLS-DA plot (VIP>1.2), p value: p values obtained after performing *t*-test (p-value<0.05), FDR: value obtained after performing false discovery test, FC: fold change (FC>1.4), AUC: area under the curve value.

#### IDC against benign

Furthermore, the metabolomic alterations that discriminate IDC subjects from benign were also screened. MVA plots for IDC and benign are shown in Supplementary Figure 5. In IDC against benign analysis, the combined results from multivariate and univariate analysis revealed 37 significantly altered metabolites (18 from LC-MRM/MS and 19 from GC-MS) (Supplementary Table 1). Among these 37 significant metabolites, 25 were with increased concentration, while remaining 12 showed decreased concentration in IDC against benign (Supplementary Table 1).

#### IDC against both control and benign

After exploring the metabolomic alterations specific to IDC within the two group analysis, our next aim was to identify the metabolomic changes discriminating IDC from both benign and control subjects. We had performed the comparison of metabolites obtained from two group analysis and scrutinized the metabolites following the pattern of increment and/or decrement from control and benign to IDC. We have observed 8 metabolites that followed the pattern of progressive change (increase or decrease) from control, benign and IDC which are mentioned in Table 3a. Histidine, glutamine, tyrosine, creatine, phenylalanine, lactic acid, and adonitol showed an elevated concentration and 3,7-cholest-5-ene showed reduced concentration from control to benign to IDC. Histidine, glutamine, tyrosine, and creatine came up as the top four metabolites showing significant alterations in tissue for this comparative study. Their concentration differences along with the ROC curve analysis is illustrated in Supplementary Figure 6.

**Table 3:** Metabolites differentiating IDC from both benign and controls a) Tissue metabolites, b) Serum metabolites

S. No	Metabolite	HMDB ID	FC (IDC/C)	AUC (IDC/C)	FC (IDC/B)	AUC (IDC/B)
**a) Tissue**						
1	Histidine	HMDB00177	3.28	0.79	2.29	0.64
2	Glutamine	HMDB00641	4.60	0.87	2.38	0.73
3	Tyrosine	HMDB00158	8.73	0.96	3.19	0.83
4	Creatine	HMDB00064	4.32	0.85	2.36	0.72
5	Phenylalanine	HMDB00159	3.98	0.92	2.45	0.81
6	Lactic acid	HMDB00190	4.35	0.85	2.54	0.76
7	Adonitol	HMDB00508	2.22	0.78	1.64	0.70
8	3,7-Cholest-5-ene	HMDB00941	0.48	0.81	0.35	0.76
**b) Serum**						
1	Ascorbic acid	HMDB00044	0.31	0.98	0.32	0.73
2	Tryptophan	HMDB00929	0.49	0.93	0.62	0.78
3	Tyrosine	HMDB00158	0.61	0.89	0.68	0.74
4	Phenylalanine	HMDB00159	0.57	0.91	0.66	0.76
5	Uric acid	HMDB00289	0.60	0.88	0.63	0.79
6	α-Ketoglutaric acid	HMDB00208	15.26	0.81	5.14	0.74
7	UDP	HMDB00295	0.43	0.91	0.46	0.83
8	Creatine	HMDB00064	0.53	0.83	0.63	0.76
9	Pyruvate	HMDB00243	0.31	0.85	0.40	0.78

HMDB ID: Metabolite ID obtained from HMDB database, FC: Fold change (FC>1.4), IDC/C: IDC against control, AUC: Area under the curve, IDC/B: IDC against benign.

### Serum metabolomic profiling

It is believed that systemic metabolomic changes in tumour tissues may be reflected by changes in biofluids such as peripheral blood. With this hypothesis, metabolomic profiles of serum samples were obtained to identify IDC specific metabolomic alterations and associate these findings with the tissue metabolomic analysis. Additionally, acquiring serum metabolomic profile of IDC would be helpful for the identification of less invasive diagnostic and prognostic markers for IDC.

#### Targeted LC-MRM/MS

**Figure 5:**
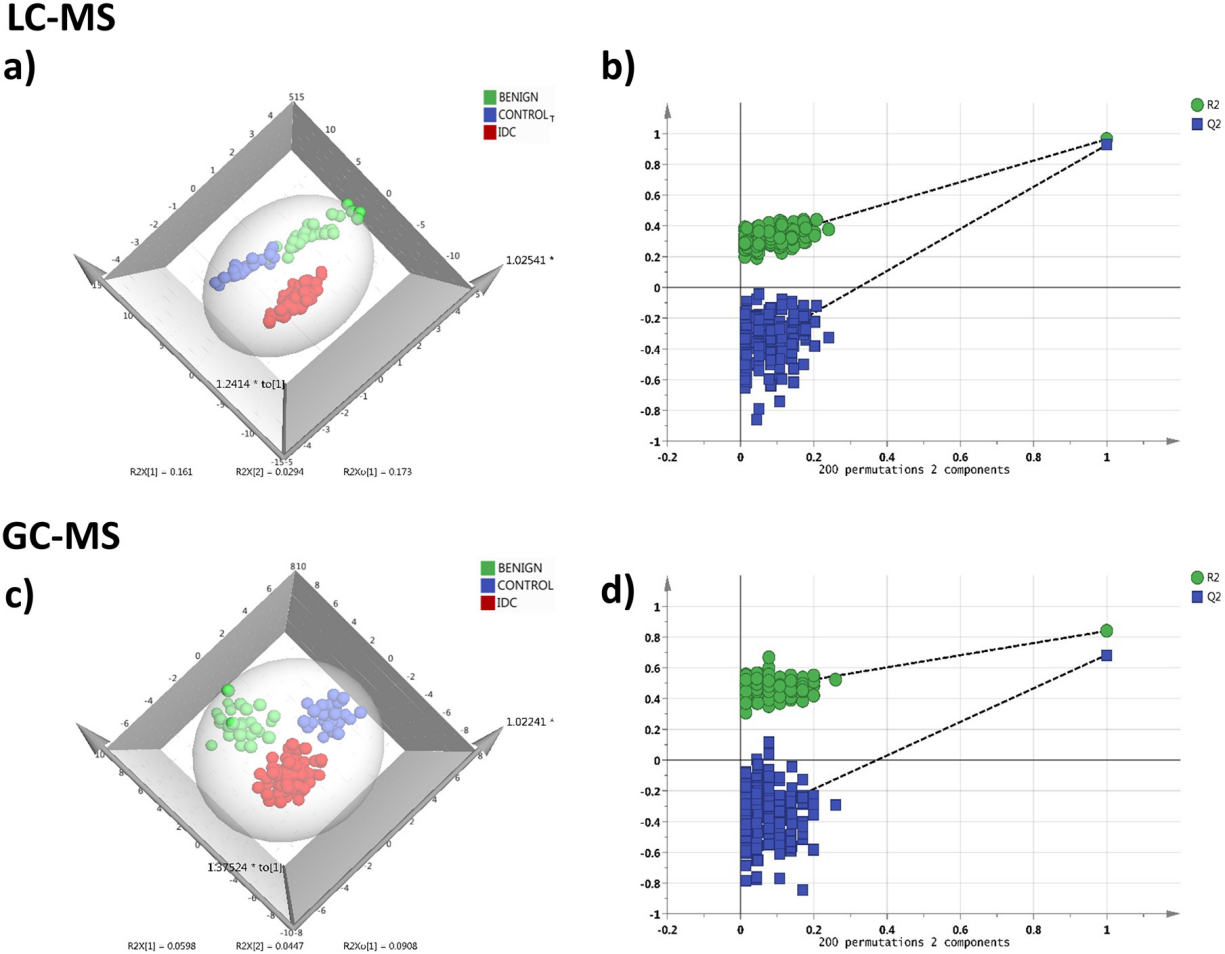
Serum metabolomics multivariate analysis LC-MRM/MS: **(a)** OPLS-DA score plot of serum LC-MRM/MS data for IDC subjects (n = 76, red), benign breast patients (n = 33, green) and healthy controls (n = 33, blue), **(b)** plot obtained after performing a random permutation test with 200 permutations on OPLS-DA model (R2Y = 0.83, Q2 = 0.71), R2 is the explained variance, and Q2 is the predictive ability of the model. Low value of R2Y and Q2-intercepts at 0.27 and -0.43 depicts the high predictability of the model, GC-MS: **(c)** OPLS-DA score plot of serum GC-MS data for IDC subjects (n = 76, red), benign breast patients (n = 33, green) and healthy controls (n = 33, blue), **(d)** plot obtained after performing a random permutation test with 200 permutations on OPLS-DA model (R2Y = 0.82, Q2 = 0.65).

In LC-MRM/MS analysis, 85 metabolites were detected at quantifiable level with minimum 30% base peak intensity. Data pre-processing was performed using median normalization, cube root transformation followed by data scaling using the feature range. Distinct clustering between the three groups IDC, benign and control was observed in OPLS-DA model (R2 0.83 and Q2 0.71) and is illustrated in Figure 5a. Cross-validating permutation test depicts validity of the OPLS-DA model (Figure 5b). HCA analysis showing group clusters is represented in Supplementary Figure 7a.

#### Untargeted GC-MS analysis

Through the GC-MS analysis of serum, 218 metabolites were detected out of which 149 had an occurrence frequency of at least 80% among the samples. The peak area data matrix was sum normalized, cubic root transformed and then auto-scaled. Clear discrimination between the three groups IDC, benign and control was observed from the OPLS-DA analysis (R2 0.82 and Q2 0.65) (Figure 5c). Permutation analysis is depicted in Figure 5d. HCA plot is shown in Supplementary Figure 7b.

### Identification of significantly altered metabolites in serum IDC against control

Similar to tissue metabolomics analysis, metabolomic alterations were examined within three groups for serum samples as well. In a comparative analysis of IDC against control, MVA showed clear discrimination between IDC subjects and healthy controls (Supplementary Figure 8). Significant alterations of 32 metabolites were observed, including 17 upregulated metabolites and 15 downregulated metabolites in IDC when compared with controls (Supplementary Table 2). A heat map of these 32 differentially regulated metabolites is shown in Supplementary Figure 9.

#### IDC against benign

Further, the IDC against benign MVA (Supplementary Figure 10) identified significant alterations of 35 metabolites, which included 15 upregulated and 20 downregulated metabolites in serum of IDC as compared to benign (Supplementary Table 3).

#### IDC against both control and benign

Finally, we investigated serum metabolites those were able to discriminate IDC samples from both control and benign samples. The metabolites that followed a pattern of increment or decrement changes in serum are listed in Table 3b. A total of 9 metabolites were identified that followed this differential regulation pattern. Ascorbic acid, tryptophan, tyrosine, phenylalanine, uric acid, UDP, creatine, and pyruvate showed reduced concentration in IDC against benign and control. Only one metabolite α-Ketoglutaric acid followed the pattern of increased concentration in IDC compared with benign and control. On the basis of VIP score, Ascorbic acid, tryptophan, tyrosine, and phenylalanine were identified as the top four metabolites showing significant alterations in serum for this three group comparative study. Their concentration differences along with the ROC curve analysis is illustrated in Supplementary Figure 11.

### Common metabolomic alterations observed in tissue and serum samples

**Figure 6:**
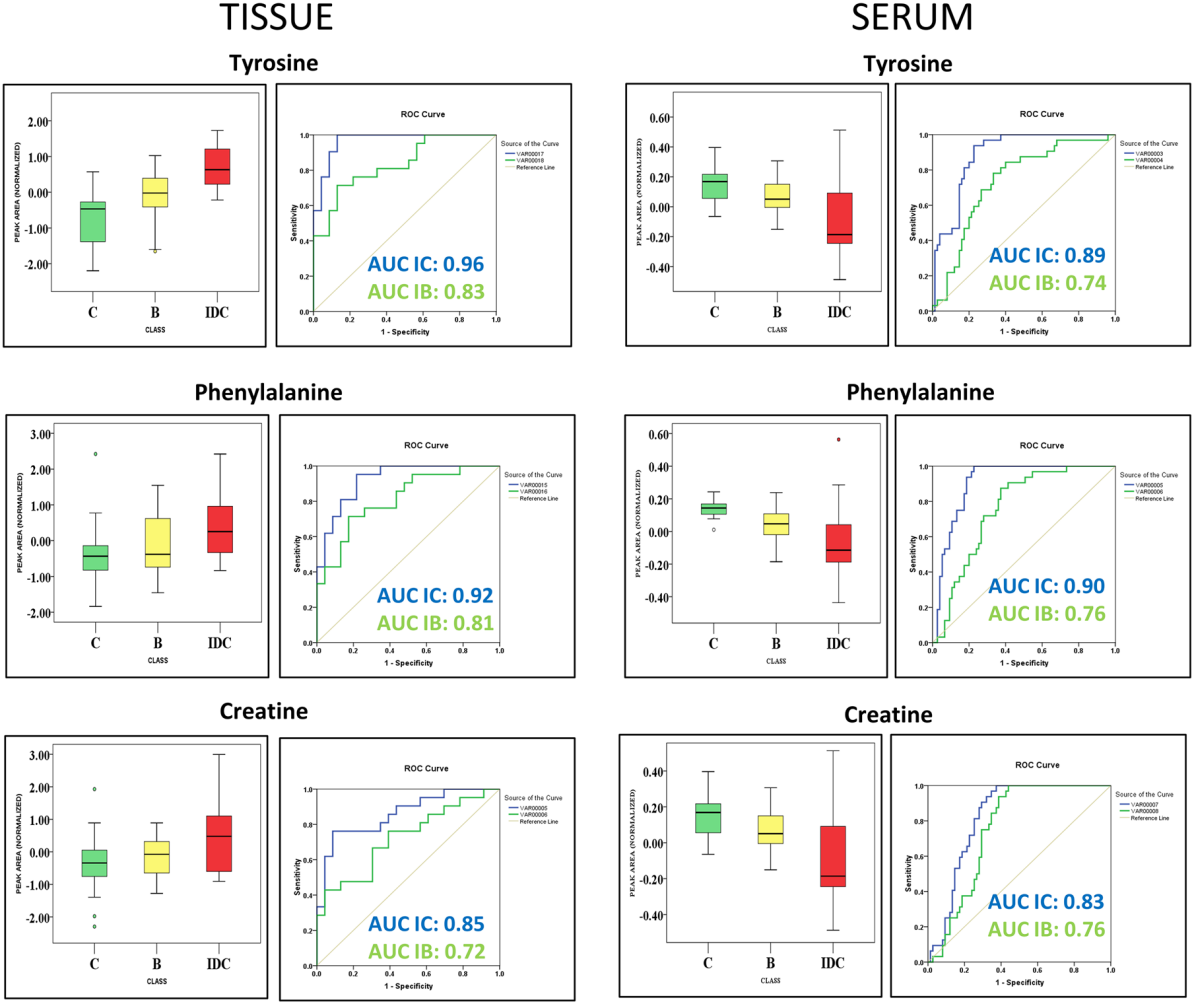
Common metabolites identified in tissue and serum discriminating IDC samples from both benign samples and healthy controls Box-and-whisker plots illustrating normalized concentration differences between control (C-green box), Benign (B-yellow box) and Invasive ductal carcinoma (IDC-red box) along with ROC curve analysis plot of sensitivity versus specificity for the three metabolites predicting IDC samples against control (IC-blue line) and IDC samples against benign samples (IB-green line). The plot depicts higher discriminative ability of these metabolites for IDC samples than benign samples.

In anticipation of identifying metabolomic changes that might be reflected from tumour tissue to serum, we screened for common metabolomic alterations identified in tissue and serum samples. In an attempt to simplify the analysis only significant metabolites from IDC against control comparison were primarily focused. We have successfully identified 10 differentially altered metabolites which were common in tissue and serum samples. The list of these common metabolomic alterations along with their concentration levels in tissue and serum is summarized in Table 4. Pentanoic acid, 11-eicosenoic acid, pentaenoate showed increased while UDP showed decreased abundance in both tissue and serum samples of IDC when compared to control. Interestingly, higher concentrations of tyrosine, phenylalanine, creatine, histidine, phosphoric acid, and ascorbic acid were observed in IDC tissue samples in comparison with control (Table 4). In contrast, lower concentrations of these metabolites were observed in IDC serum when compared with control (Table 4). Additionally, from the three group analysis, we have observed tryptophan, tyrosine, and creatine as common metabolites present in both tissue and serum. Interestingly, the levels of tryptophan, tyrosine, and creatine in tissue are in negative correlation with that of serum (Table 4). Further, these metabolites were able to discriminate IDC subjects from both benign and control subjects in tissue and serum samples (Figure 6). These 3 were the only metabolites that showed a pattern of progressive change (either increase or decrease) from control to benign to malignant (IDC) individuals in both tissue and serum samples.

**Table 4:** Common metabolites from tissue and serum differentiating IDC from controls

S. No	Metabolite	HMDB ID	FC TISSUE	FC SERUM
1	11-Eicosenoic acid	HMDB34296	51.94	2.53
2	Pentanoic acid	HMDB00892	11.99	2.78
3	Phosphoric acid	HMDB02142	10.72	0.46
4	Tyrosine	HMDB00158	8.73	0.61
5	Pentaenoate	HMDB00290	4.55	1.63
6	Creatine	HMDB00064	4.32	0.53
7	Phenylalanine	HMDB00159	3.98	0.57
8	Histidine	HMDB00177	3.28	0.50
9	Ascorbic acid	HMDB00044	3.07	0.31
10	UDP	HMDB00295	0.59	0.43

HMDB ID: Metabolite ID obtained from HMDB database, FC: fold change (FC>1.4).

### Pathway analysis

**Figure 7:**
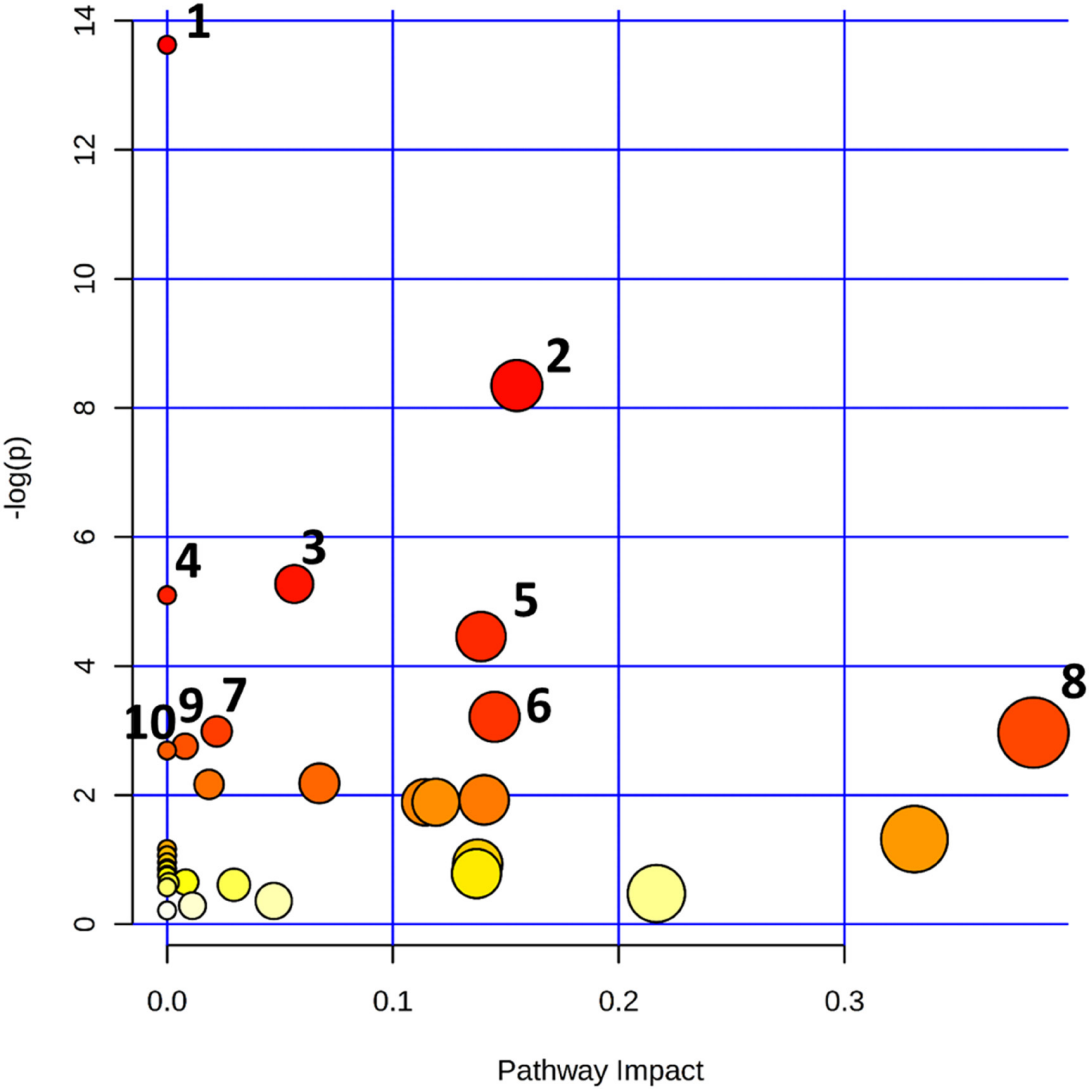
Altered metabolomic pathways observed in tissue of IDC Topology map of differentially expressed metabolites generated using metaboanalyst describing the impact of metabolites identified through comparative analysis of IDC against control. Top pathways identified in tissue are 1. Nitrogen metabolism, 2. Pyrimidine metabolism, 3. Aminoacyl-tRNA biosynthesis, 4. Fatty acid biosynthesis, 5. D-Glutamine and D-glutamate metabolism, 6. Riboflavin metabolism, 7. Purine metabolism, 8. Alanine, aspartate and glutamate metabolism, 9. Phenylalanine, tyrosine and tryptophan biosynthesis, 10. Beta-Alanine metabolism.

The statistically significant metabolites discerned from the comparative analysis of IDC against control samples were subjected to pathway analysis in order to elucidate the metabolic pathways perturbed in IDC. The results obtained from the pathway analysis of the significantly altered metabolites from tissue are illustrated in Figure 7. The top ten pathways observed in tissue samples are nitrogen metabolism, pyrimidine metabolism, aminoacyl-tRNA biosynthesis, fatty acid biosynthesis, D-glutamine and D-glutamate metabolism, riboflavin metabolism, purine metabolism, alanine, aspartate and glutamate metabolism, phenylalanine, tyrosine and tryptophan biosynthesis, and beta-Alanine metabolism. Detailed pathway analysis table including all the identified pathways is given in Supplementary Table 4.

## DISCUSSION

Until now metabolomics studies in breast cancer has been mainly focused to understand the metabolomic changes in serum and plasma samples [21–23]. The complementary approach of screening a marker primarily in tissue first, followed by its validation in bofluids is described in few proteomics based studies [31, 32]. However, the same has not been implemented yet in metabolomics of breast cancer. Although the likelihood of getting all the changes from tissue in serum is limited as the blood gets contribution from all the tissues in body. However, a complex interplay between tumour tissue and the surrounding microenvironment may have substantial role in the alteration of serum metabolic profile, necessitating the need of simultaneous tissue and serum metabolomic profiling. Another important yet debatable aspect in breast cancer progression is the benign to malignant transformation of a tumour [26]. Metabolomics offers unique opportunity to delve deep inside the regulation of pathways underlying various biological processes, which, in-turn can provide valuable information about the benign to malignant transformation of breast tumour. In addition, herein two metabolomics approaches (targeted and untargeted) were employed to generate a comprehensive metabolomics profile of IDC. Although both approaches are complementary, increased compound detection and throughput compound identification in GC-MS gives it an edge over the targeted LC-MS. The increased metabolomic coverage leads to detection of broad range of metabolites. This may help to generate the holistic view of key metabolic pathway alterations in IDC, eventually improving the present clinical approach for IDC.

The present study, for the first time, outlines the tissue and serum metabolomic profiles of IDC using both LC-MRM/MS and GC-MS; differentiating them from benign and control samples. The key findings of this study include the identification of 42 significant metabolites consisting of 36 upregulated and 6 downregulated metabolites in IDC tissue against control. Additionally, 32 significant metabolites, including 17 upregulated and 15 downregulated metabolites were identified in IDC serum compared to control. These metabolites includes amino acids, nucleotides and nucleosides, fatty acids, amino sugars, organic acids, vitamins and other organic compounds. Moreover, reciprocal metabolomic alterations were observed between tissue and serum, indicating a bi-directional interaction of metabolites in IDC. Interestingly, 8 tissue metabolites and 9 serum metabolites showed progressive change from control to benign to IDC suggesting their possible role in malignant transformation. Finally, we have identified a panel of 3 metabolites viz. tryptophan, tyrosine, and creatine in both the tissue and serum, which could be useful in screening of IDC subjects from control as well as benign patients. Since, this study included multiple groups, to avoid the ambiguity in discussion, only metabolomic alterations observed in IDC against control with primary focus on tissue and some of the concomitant alterations observed in serum are further discussed.

The 42 significantly altered metabolites in IDC tissue as compared to controls were mapped to several important pathways. The most important pathways observed to be altered in tissue were nitrogen metabolism, pyrimidine metabolism, aminoacyl-tRNA biosynthesis, fatty acid biosynthesis, D-glutamine and D-glutamate metabolism, riboflavin metabolism, purine metabolism, alanine, aspartate and glutamate metabolism, phenylalanine, tyrosine and tryptophan biosynthesis, beta-alanine metabolism, arginine and proline metabolism, and glutathione metabolism (Figure 7). The metabolic network map reflects the important interactions between the altered metabolic pathways (Figure 4). Notably, an increase in amino acid metabolism comprising increase in tyrosine, phenylalanine, glutamic acid, glutamine, and histidine was observed (Table 2). These findings are in accordance with previous reports where an increase in amino acid turnover in breast cancer is reported [33]. In contrast, lower concentrations of various amino acids were observed in IDC serum, which is in agreement with previous report (Supplementary Table 2) [34]. The diminished concentrations of the amino acids in IDC serum may be attributed to their higher uptake by the tumour tissue to fulfil the proliferative demand [34].

Two other important metabolic pathways found to be altered in IDC were purine metabolism and pyrimidine metabolism (Figure 4). Proliferating tumour cells often demand for nucleotides for the rapid synthesis of cellular materials, which is fulfilled by de novo synthesis and/or nucleotide salvage of purines and pyrimidines [35]. Nucleotide salvage pathway uses degraded nuclear material (DNA/RNA) in the form of purines and pyrimidines to reproduce the dNTPs for DNA/RNA synthesis [35]. Besides, increased concentration of a modified nucleotide 7-methylguanosine in IDC tissues was also observed which is suggestive of its demand for the increased ‘cap’ formation for rapid protein synthesis in IDC [36]. Similarly, IDC serum showed elevated concentration of nucleotides including CTP and UTP, which may be attributed to their release from damaged cells. The extracellular nucleotides are also known to stimulate the cell survival, proliferation and migration through purinergic signaling [37]. Interestingly, lower levels of cyclic AMP, a negative regulator of epidermal growth factor receptor (EGFR) is observed in IDC serum, thereby possibly providing survival advantage for tumours and promote further growth [38].

Additionally, fatty acid metabolism is indicated to be alerted in IDC tissue. Specifically, increased levels of numerous unsaturated fatty acids and decreased level of saturated fatty acids in IDC tissues were observed (Figure 4). The elevated unsaturated fatty acid levels helps in increasing the membrane fluidity and modulation of adhesion [39]. These findings are also in accordance with our previous report in breast cancer, where an increase in phospholipids with unsaturated fatty acid composition is reported [40]. An increase in the concentration of omega-6-polyunsaturated fatty acids (PUFAs) has been observed, which are important in modulating various physiological functions including initiation and sustainment of inflammation (Figure 4) [41]. Moreover, the increased fatty acid metabolism has also been reported to be associated with invasive breast cancer [42].

Amino sugar metabolism (hexosamine pathway) is also observed to be altered in the IDC tissue (Figure 4). These observations are in good agreement with a recent breast cancer metabolomics study by Hadi et al. using GC-MS [42]. N-acetylgalactosamine (GalNAc) is an important component of glycoprotein as it is the first glycan added in the O-glycosylation process, initiation of which aids invasiveness [43]. Another amino sugar N-acetylglucosamine (GlcNAc) is reported to mediate cell signaling directly and indirectly, promoting tumour cell invasion [44]. Riboflavin metabolism generates flavin moieties, which are important players in cellular metabolism. The increase in riboflavin uptake and its metabolism in tumour tissue, including breast cancer, has already been reported [45]. The increased levels of lactic acid along with carbonic acid confirms the activity of glycolytic switch leading to decrease in the pH of tumour, ultimately helping tumour cell in immune disruptions [46, 47]. The carbonic acid increase corroborates with previous report where higher expression of carbonic anhydrase, an enzyme involved in conversion of carbon dioxide to carbonic acid is reported [48].

In addition, we have observed 10 altered metabolites, which are common in both tissue and serum. Among them, 4 showed similar concentration change in both tissue and serum, while the concentration change in other 6 metabolites were opposite (Table 4). Increase in fatty acids in both serum and tissue is indicative of their de-novo synthesis in serum and uptake in tissue to support proliferation [49]. As discussed above, the lower levels of the amino acids in IDC serum could be due to their increased uptake by the tumour tissue leading to their accumulation in tissue and deprival in serum [34]. Other important metabolic changes shared by both tissue and serum were involved in antioxidant machinery, energy metabolism etc. [50–52]. Special attention was necessary for the metabolites that showed a pattern of progressive change from control to benign to malignant IDC as they may possibly be involved in benign to malignant tumour transformation. These metabolites include histidine, glutamine, tyrosine, creatine, phenylalanine, lactic acid, adonitol, and 3,7-cholest-5-ene. As discussed earlier, these metabolites are important players in energy metabolism, cell signaling processes, cancer progression and development processes which are under the direct or indirect control of oncogenes. These metabolic alterations needs to be explored further in context to identify their possible roles in benign to malignant transformation.

A panel of three metabolites including tryptophan, tyrosine, and creatine may prove to be helpful in discrimination of IDC subjects from both control and benign. Only these 3 metabolites showed a pattern of progressive change (either increase or decrease) from control to benign to malignant (IDC) individuals in both tissue and serum sample. Interestingly, a reverse trend of progressive metabolic change, an increase in tissue while decrease in serum, was observed for these metabolites. As discussed above, amino acids (tryptophan and tyrosine) increase in IDC patients, is suggestive of their preferred demand in tissue [34]. This demand is fulfilled by their preferred uptake and utilisation in tissue, leading to their decreased levels in serum [34]. Further, the lower levels of creatine in serum indicates its possible conversion to phosphocreatine by creatine kinases that serve as an energy reserve [52]. This phosphocreatine might have been taken up by tumour tissues then dephosphorylated back to creatine to fulfil the energy demand [51]. Interestingly, the higher activity of creatine kinases in serum is already reported indicating its role in invasive breast cancer [51]. Overall, this study provides valuable insights into the metabolic adaptations of IDC tumours. However, certain limitations associated with this study needs to be considered. First, an extensive validation study using a large cohort of subjects needs to be performed in order to confirm the findings of our study. Second, there is a possibility that different molecular subtypes of IDC may have an effect on the metabolomic profile. Hence, it will be interesting to look for the metabolomic alterations in the molecular and histological subtypes of IDC in the future.

In summary, this study is the first attempt to understand comprehensive metabolomic alterations specific for IDC of the breast. We have demonstrated that the metabolomic alterations exist in tissue and serum samples of the subjects with IDC, differentiating them from healthy controls and benign subjects. Some of the metabolic changes from tissue were also reflected in serum, emphasizing the bi-directional interaction between blood and tumour tissue. The results suggest possible role of several metabolic pathways in IDC including amino acid metabolism, purine and pyrimidine metabolism, fatty acid metabolism, amino sugar metabolism. In our ongoing work, we are focusing on to decipher the mechanistic role of these pathways in IDC progression. In addition, it was noteworthy to identify the progressive metabolomic alterations in control to benign to malignant IDC breast conditions. In future, with this lead, it will be interesting to understand their possible role in benign to malignant transformation. Nevertheless, the results presented in this study can serve as useful resource for identification of potential targets from which new therapeutic agents might be developed.

## MATERIALS AND METHODS

### Subject selection and sample collection

Subjects were voluntarily recruited with written informed consent at Ruby Hall Clinic Cancer Centre, Pune during the period of May 2013 to June 2016. Ethical approval was obtained from the Ethics Committees of Poona medical research foundation and National Centre for Cell Science (NCCS). Subjects with cancerous breast tumour were considered as malignant while non-cancerous breast tumour subjects were considered as benign. IDC status of malignant tumours was confirmed by histopathological analysis. Clinical specimens, tissue and serum for IDC and benign groups were collected from the same subjects at the time of the surgery. The tumours selected were appropriately stage and size matched according to new TNM staging system. Normal tissue as a control was obtained from IDC tumour adjacent tissue (2-5 cm away). Control serum samples were obtained from the subjects undergoing breast health check-up at the camp organized by the hospital. Detailed description of sample collection including inclusion and exclusion criteria of subjects is given in supplementary materials and methods. The overall histological characteristics of the patients included in this study are given in Table 1.

### Targeted LC-MRM/MS metabolomic profiling

Serum metabolite extraction was performed by adding 400 μl of ice-cold methanol to 50 μl of serum. In case of tissue, samples (50 mg) were homogenized in 400 μl of ice-cold methanol using Precellys homogeniser (Bertin Corp, USA) and metabolites were extracted. (Details in supplementary material and methods). Prior to extraction 2 μl of internal standard d_2_ L-phenylalanine (100 ng/μl) was added. For targeted metabolomic profiling, 108 metabolite standards were selected based on the literature survey of cancer metabolomics studies. The analysis was performed using multiple reaction monitoring (MRM) mode on a 4000 QTRAP triple quadrupole mass spectrometer (AB SCIEX, Foster City, CA) equipped with Shimadzu Prominence binary pump HPLC (Shimadzu Corporation, Japan). During positive ionization mode, the dried metabolite extract was dissolved with 50 μl solvent (6.5:2.5:1 acetonitrile: methanol: water) and injected on XBridge HILIC column (Waters, Milford, MA). In the negative ionization mode, metabolites extract was dissolved in 50 μl ultrapure water and injected on ATLANTIS T3 column (Waters, Milford, MA). For both modes, 10 μl of the sample was injected using the HPLC autosampler. Data analysis was performed in the Analyst 1.5 software (Sciex, Foster City, CA). The sample orders were randomized at the time of analysis. Internal standard peak areas were used to evaluate the metabolite extraction efficiency as well as to check the instrument performance over the time. Metabolites with a minimum of 30% of base peak intensity and consistently detected in 80% of the samples were considered for quantitation. Further, the peak areas obtained after integration were exported to a spreadsheet file in a matrix format. The resulted data sets were subjected to statistical analysis.

### Untargeted GC-MS metabolomic profiling

For GC-MS analysis, 50 mg tissue and 50 μl serum with internal standard 2- isopropyl-malic acid (15 μl from 1.0 mg/mL) was taken and metabolites were extracted similar to LC-MS. Two step derivatization was performed using methoxyamine hydrochloride in pyridine followed by BSTFA with 1% TMCS. After derivatization 1 μl of the sample was injected into an Agilent 5975C GC system (Agilent Technologies Inc., USA). HP-5 MS ultra-inert fused silica capillary column (30 m × 0.25 μm × 0.25 mm, Agilent, USA) was used for the chromatographic separation of metabolites. The column effluent was introduced into the ion source of an Agilent 5977 mass selective detector (Agilent Technologies Inc., USA). GC-MS metabolite profiles were processed using Agilent Chemstation^TM^ data analysis software. The sample runs were randomised at the time of analysis. The metabolite extraction efficiency and instrument performance over the time was evaluated by using peak area of internal standard. Metabolite identification was performed by comparing the mass fragmentation pattern with NIST 11 Standard mass spectral databases in NIST MS search 2.0 (NIST, Gaithersburg, MD) software. Consistent metabolites with minimum 30% base peak intensity were considered for quantitation. The peak areas obtained after integration were exported to a spreadsheet file in a matrix format. Further the dataset was subjected to statistical analysis.

### Multivariate statistical analysis

LC-MRM/MS and GC-MS metabolomics data from tissue and serum samples were pre-processed using Metaboanalyst 3.0 [53]. For LC-MS, peak area data matrix was pre-processed using sample median normalization, cube root transformation, and range scaling methods. While in case of GC-MS the data matrix was sum normalized, cubic root transformed and then auto-scaled. The pre-processed data files were subjected to multivariate analysis using SIMCA 14 software (Umetrics, Sweden). Orthogonal partial least squares discriminant analysis (OPLS-DA) was employed to identify the group separation [54]. Goodness of the fit and predictive ability of the OLPS-DA models are assessed by R2 and Q2 values [55]. Further, cross-validation using 200 permutations were performed to avoid overfitting of the model. Additionally, hierarchical cluster analysis (HCA) was performed to identify group clusters.

### Significant metabolites selection

Significant variables for group separation in OPLS-DA model were identified using variable importance in the projection (VIP) score. Variables having VIP score value above 1.2 were considered important for discrimination. Student's *t*-test statistics were performed and significant metabolites (p < 0.05) were adjusted for multiple hypothesis testing using FDR correction. Metabolites having fold change threshold of 1.4 and above were considered significant. Metabolites collectively qualifying the criteria of VIP, p-value, FDR, and fold change were considered significant. All the univariate analyses were performed using Metaboanalyst 3.0. Box-and-whisker plots and ROC curve analysis plots were plotted using SPSS 20.0. Heat maps of differentially expressed metabolites were created using Multi-Experiment Viewer software [56].

### Pathway analysis

The differentially expressed metabolites from the comparative analysis of IDC against control were further subjected to pathway analysis using a pathway analysis tool (MetPA) in Metaboanalyst 3.0 [53, 57]. The Pathway Analysis module in Metaboanalyst puts together the results obtained from pathway enrichment analysis and pathway topology analysis to unravel the most relevant pathways involved in the study condition. In addition, it uses information from database sources, including the Kyoto encyclopedia of genes and genomes (KEGG) [58] and human metabolome database (HMDB) [59] for the identification of affected metabolic pathways. Although pathway analysis generates valuable information, the inferences drawn from it should be dealt with the caution, as some of pathway inferences from metabolomics study can be based on one or two metabolites only.

### Data accessibility

Metabolomics data have been deposited to the EMBL-EBI MetaboLights database with the identifier MTBLS552 [60]. The complete dataset can be accessed here https://www.ebi.ac.uk/metabolights/MTBLS552

## SUPPLEMENTARY MATERIALS FIGURES AND TABLES










