# Supplementary material for: Metabolomic alterations in invasive ductal carcinoma of breast: A comprehensive metabolomic study using tissue and serum samples
Source: Oncotarget. 2017 Dec 23;9(2):2678–96. doi: 10.18632/oncotarget.23626 (PMC5788669; doi:10.18632/oncotarget.23626)
Supplement: Supplementary file 1 [file oncotarget-09-2678-s001.pdf]

Metabolomic alterations in invasive ductal carcinoma of breast: A comprehensive metabolomic study using tissue and serum samples

SUPPLEMENTARY MATERIALS

Subject selection

Clinical specimens, tissue and serum for the study were collected from the subjects who underwent modified radical mastectomy (MRM) or breast biopsy at Ruby Hall Clinic Cancer Centre, Pune during the period of May 2013 to June 2016. The study was ethically approved by hospital Ethics Committee of Poona medical research foundation along with the institutional ethics committee of National Centre for Cell Science (NCCS). Subjects were voluntarily recruited with written informed consent as per the guidelines of Helsinki's Declaration (2008). The study subjects were freshly diagnosed breast cancer patients who didn't undergo any therapeutic intervention such as neoadjuvant chemotherapy or radiotherapy. Subjects with a non-cancerous tumor in the breast were recruited as benign. Malignant IDC or benign status of breast tumors was confirmed by histopathological analysis of tumors. Control tissue samples used for the study was obtained from the tissue adjacent (5 cm away) to tumor which was collected from the same patient undergoing the mastectomy. Control serum samples were obtained from the subjects undergoing breast health check-up at the camp organized by the hospital. These subjects were confirmed for not having any lesions in the breast after physical examination followed by mammography of the breast. The inclusion criteria for the healthy subjects were as follows, subjects who had no diabetes, hypertension and have not had any medical and hormonal supplementation given during the last 6 months. The overall histological characteristics of the patients included in this study are given in Table 1.

Sample collection

Subjects were asked to undergo an overnight fasting and subsequently on the next day venous blood samples were collected. The blood samples were allowed to clot at room temperature and then centrifuged at 1000g for 10 min. The clear sera were then transferred into sterile vials, snap frozen and stored at -80°C until further analysis. In case of tissue samples, the tumor and normal tissue were collected immediately after surgery and put in the cryovials which were then transported in -20°C mini cooler from the hospital to the experimental laboratory. Tissue samples were then snap frozen and stored in -80°C ultra-low temperature freezer for long term preservation.

Targeted LC-MRM/MS metabolomic profiling

Sample preparation

Serum: Metabolite extraction was performed using adding 400 µl of ice-cold methanol to 50 µl of serum spiked with 2µl of internal standard. This mixture was vortexed and kept overnight incubation at -20°C for deproteination. Samples were then vortexed and centrifuged at 14,000g for 10 min at 4°C. The supernatant was collected and filtered through 0.22 µm nylon filter. The filtrate was collected and dried in SpeedVac.

Tissue: Tissue samples weighing 50 mg was taken in homogeniser vial containing zirconium beads. 400 µl of ice-cold methanol spiked with internal standard d_2 L-phenylalanine was added to homogenizer vial. Tissue homogenization was carried out using Precellys homogeniser (Bertin Corp, USA). The following homogenizer program was used: 2 cycles of 6000 rpm for 20 sec and 2 cycles of 6500 rpm for 30 sec, with intermittent cooling on ice between each cycle. The homogenizer vials were then centrifuged at 14,000 rpm for 10 min at 4°C. The supernatant was collected and filtered through 0.22 µm Nylon filter. The filtrate was transferred to the vial and evaporated to dryness using SspeedVac.

The dried metabolite extract from serum and tissue were dissolved in 50 µl sample buffer (6.5:2.5:1 Acetonitrile: Methanol: Water) and used for positive ionization mode (HILIC Chromatography). In case of negative ionization mode, the dried metabolite extract from serum and tissue were dissolved in 50 µl ultrapure water (T3 RPLC Chromatography). For both modes, 10 µl of the sample was injected using an autosampler.

LC-MRM/MS analysis

Targeted metabolomic analysis was performed using multiple reaction monitoring (MRM) based approach. Based on literature survey of cancer metabolomics studies, 108 metabolite standards were purchased from Sigma Aldrich to build an in-house MRM method. For each metabolite standard, parent ion to daughter ion transitions were selected using MS/MS fragmentation.

Based on the fragmentation pattern of metabolites, 108 metabolite standards were further divided into positive and negative ionization mode. For each MRM transition, collision energy (CE) and declustering potentials (DP) were optimised. The information obtained was exported to build the acquisition methods for positive and negative ionisation modes. In order to maximise the metabolomic coverage, chromatographic separation was performed using hydrophilic and reversed phase chromatography columns for positive and negative ionisation mode respectively. For both modes, 10 µl of sample was injected into the mass spectrometer using Shimadzu Prominence HPLC autosampler.

MS data was acquired using a 4000 QTRAP triple quadrupole mass spectrometer (AB SCIEX, Foster City, CA) equipped with Shimadzu Prominence binary HPLC pump (Shimadzu Corporation, Japan). For positive ionisation mode, the chromatographic separation was achieved using XBridge HILIC column (Waters, Milford, MA) that was eluted at 700 µL/min with a 32 min linear gradient starting from 5% mobile phase A (10 mM ammonium formate with 0.1% formic acid) increasing to 60% mobile phase B (acetonitrile with 0.1% formic acid). The column was kept at 60% mobile phase B for 3 min then returned to 5% mobile phase A for equilibration. For negative ionization mode, the chromatographic separation was achieved using ATLANTIS T3 column (Waters, Milford, MA) that was eluted at 500 µL/min with a 40 min linear gradient starting with 100% mobile phase A (10 mM ammonium hydroxide with 0.1% acetic acid) increasing to 98% mobile phase B (100% MeOH). The column was kept at 98% mobile phase B for 5 min then returned to 100% mobile phase A for equilibration. MRM was used to acquire targeted MS data for specific metabolites in the positive and negative ion modes. Analyst 1.5 software (SCIEX, Foster City, CA) was used to set transitions, dwell time and collision energies. MS conditions were set as follows, source temperature: 400°C, interface heater: on, curtain gas: 30, declustering potential: 90, entrance and exit potential: 10, and the two ion source gases were set at 45 (arbitrary units). LC-MRM/MS data was analysed by the Analyst 1.5 software (Sciex, Foster City, CA) for manual inspection of chromatograms and for the detection of the compounds. Analyst quantitation wizard was used for integration of peak areas. For quality measures, samples order were randomized at the time of analysis and integration of peaks was performed in a blinded manner. Metabolites with a minimum of 15% of base peak intensity were considered for quantitation. The peak areas obtained after integration were exported to a spreadsheet file in a matrix format for univariate and multivariate analysis.

Untargeted GC-MS metabolic profiling

Sample preparation

In case of GC-MS analysis, 50µl serum and 50 mg tissue samples, spiked with an internal standard (15 µl 2- Isopropyl-malic acid in water, 1.0 mg/mL) was taken. Metabolites extraction from serum and tissue was performed as described in LC-MS sample preparation section. Alternately, the metabolite extract was collected in a glass vial and dried under a stream of nitrogen gas. Two-step derivatization was carried out to obtain the trimethylsilyl derivatives of metabolites. At first metabolite extract was derivatized to form methylated derivatives by adding 30 µl of methoxyamine hydrochloride solution in pyridine (20 mg/ml) and incubating for 90 min at 37°C. The second derivatization was achieved by adding 30 µl BSTFA with 1% TMCS to the mixture and heated for 1 h at 70°C to yield trimethylsilyl derivatives. 1 µl aliquot of each sample is injected in the GC-MS.

GC-MS analysis

After derivatization 1 µl of the derivatized sample was injected into an Agilent 5975C GC system (Agilent Technologies Inc., USA). HP-5 MS ultra-inert fused silica capillary column (30 m x 0.25 µm x 0.25 mm, Agilent, USA) was used for the chromatographic separation of derivatized metabolites. Helium was used as carrier gas at a rate of 1 mL/min for metabolite separation within the column. The GC column inlet temperature was set at 280°C. The GC oven temperature was initially kept at 50°C for 2 min, then increased to 100°C with a ramp of 3°C/min, where it was maintained for 2 min and then increased to 200°C with a ramp of 3°C/ min, where it was maintained for 2 min. The column effluent was introduced into the ion source of an Agilent 5977 mass selective detector (Agilent Technologies Inc., USA). The MS quadrupole temperature was set at 150 °C, and the ion source temperature was set at 230°C. Data acquisition was performed in the full scan mode from m/z 50 to 550. GC-MS metabolite profiles were processed using Agilent Chemstation data analysis software. Metabolite identification from GC-MS was performed by comparing the mass fragmentations with NIST 11 Standard mass spectral databases in NIST MS search 2.0 (NIST, Gaithersburg, MD) software. Features were annotated to metabolites with a similarity of more than 90% in mass spectral pattern matching. After chromatographic integration peak areas of corresponding metabolites were noted. The peak areas obtained after integration were exported to a spreadsheet file in a matrix format to further perform statistical treatments to the data matrix.

Multivariate statistical analysis

Metabolites vary in concentration across biological samples, hence data pre-processing methods were applied in order to minimize the variance. Raw data in spreadsheet format obtained from LC-MRM/MS and GC-MS analysis were subjected to data pre-processing methods such as data normalization, data scaling, and data transformation. Data obtained from LC-MRM/MS and GC-MS analysis of serum and tissue were pre-processed independently using aforementioned data processing methods in Metaboanalyst 3.0 web server. After pre-processing data was subjected to SIMCA 14 software (Umetrics, Sweden) for multivariate analysis. Unsupervised clustering methods such as principal component analysis (PCA) were performed in order to identify the general trends and possible outliers in the data matrix. Orthogonal partial least squares discriminant analysis (OPLS-DA) was employed to identify the group separation. OPLS-DA model was further subjected to cross-validation permutation analysis using 200 permutations. In order to check the validity of model R2Y (cum) and Q2 (cum) values were calculated. The goodness of fit of the model is estimated by R2Y (cum) and the predictive accuracy of the model is estimated by Q2 (cum) values. In order to identify the clusters between the groups based on intrinsic similarities of metabolites, hierarchical cluster analysis (HCA) was also performed. Variable importance in the projection (VIP) analysis was performed on OPLS-DA model to obtain the significant variables responsible for the group discrimination.

Significant metabolites selection

Significant metabolites responsible for the group discriminations were selected using VIP score. Variables having VIP score value above 1.2 were considered important for discrimination. In order to find the metabolites responsible for two group discrimination univariate statistical analysis along with multivariate analysis was performed. For univariate statistical analysis, student's T-test statistics was performed to identify the significance of the metabolites. The significant metabolites with $p > 0.05$ were further adjusted for multiple hypothesis testing using FDR correction. Concentration differences of the metabolites were calculated by fold change calculations. Metabolites having fold change threshold of 1.4 and above were considered significant. All the aforementioned univariate statistical analyses like T-test, FDR and fold change were performed on normalized data matrix using Metaboanalyst 3.0. The final list of significant metabolites was confirmed by comparing univariate and multivariate statistical analyses. Metabolites collectively qualifying the criteria of VIP, p-value, FDR, and fold change were considered significant. Differences between concentrations of significant metabolites were shown using box-and-whisker plots. Receiver operating characteristic curve analysis (ROC) was performed to assess the predictive ability of metabolites. Box-and-whisker plots and ROC curve analysis were carried out using SPSS 20.0. In order to visualize the concentration differences of metabolites, heat maps of differentially expressed metabolites were created using Multi-Experiment Viewer software publically available at http://mev.tm4.org.

Pathway analysis

The differentially expressed metabolites from the comparative analysis of IDC against control were further subjected to pathway analysis using pathway analysis tool (MetPA) in Metaboanalyst 3.0. The Pathway Analysis module in Metaboanalyst combines results from pathway enrichment analysis with the pathway topology analysis to elucidate the most relevant pathways involved in the conditions under study. In addition, it uses information from database sources, including the kyoto encyclopedia of genes and genomes (KEGG) (http://www.genome.jp/kegg/), and Human Metabolome Database (HMDB) for the identification of affected metabolic pathways.

a)

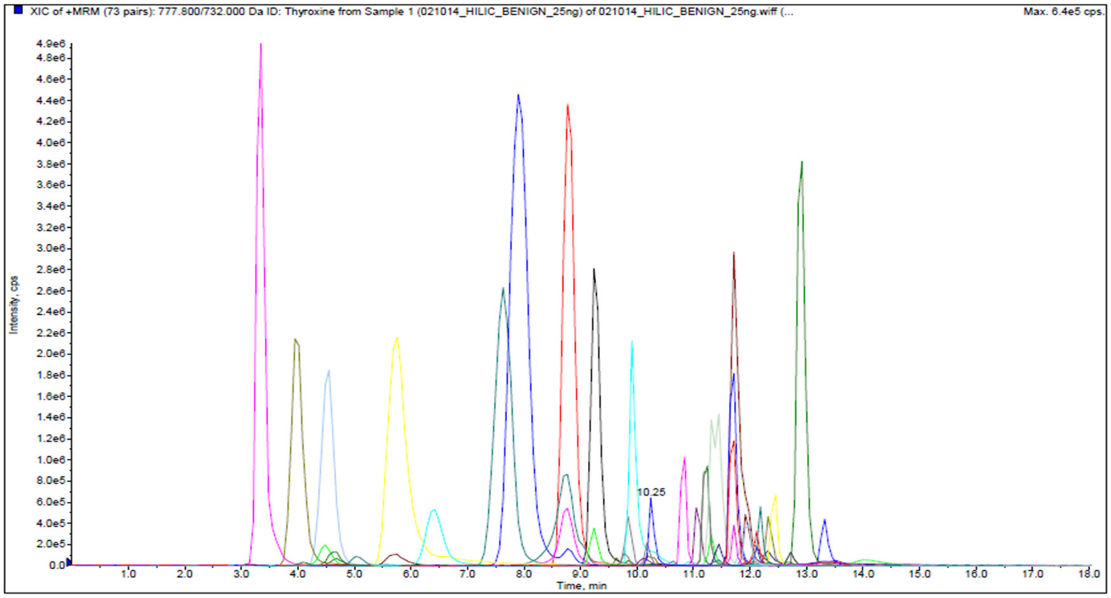

b)

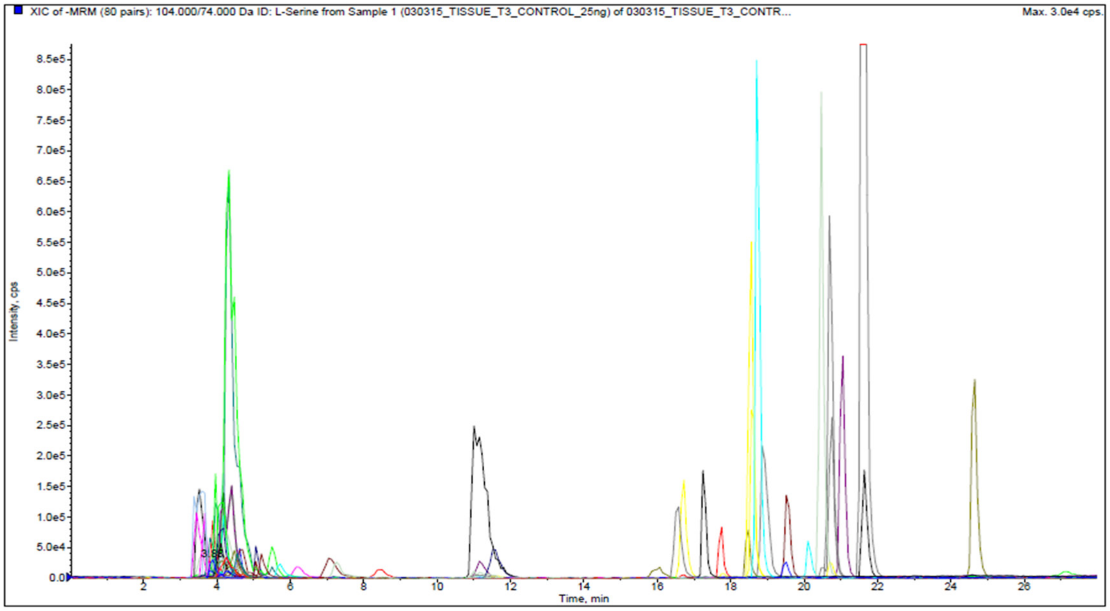

Supplementary Figure 1: Representative mass spectra of metabolites **(a)** Targeted LC-MRM/MS spectrum of metabolites obtained from HILIC analysis, **(b)** Targeted LC-MRM/MS spectrum of metabolites obtained from T3 analysis.

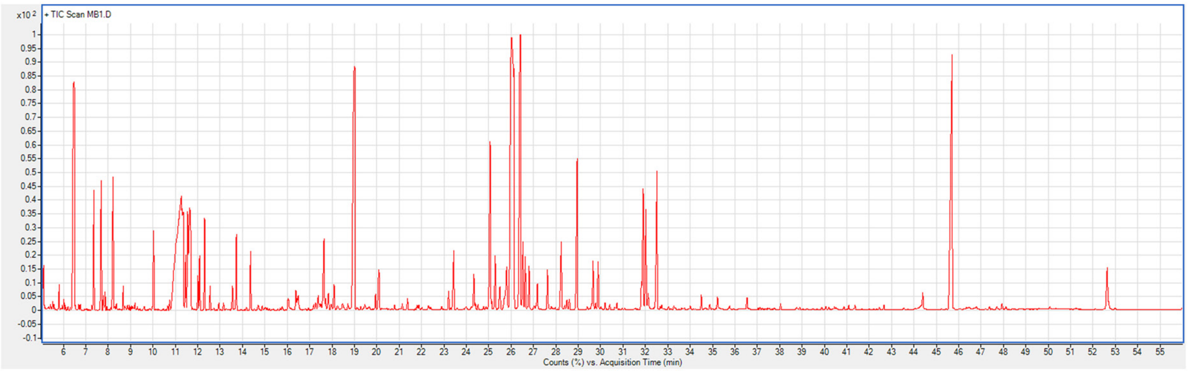

Supplementary Figure 2: Representative mass spectra of metabolite profiling obtained using untargeted GC-MS analysis.

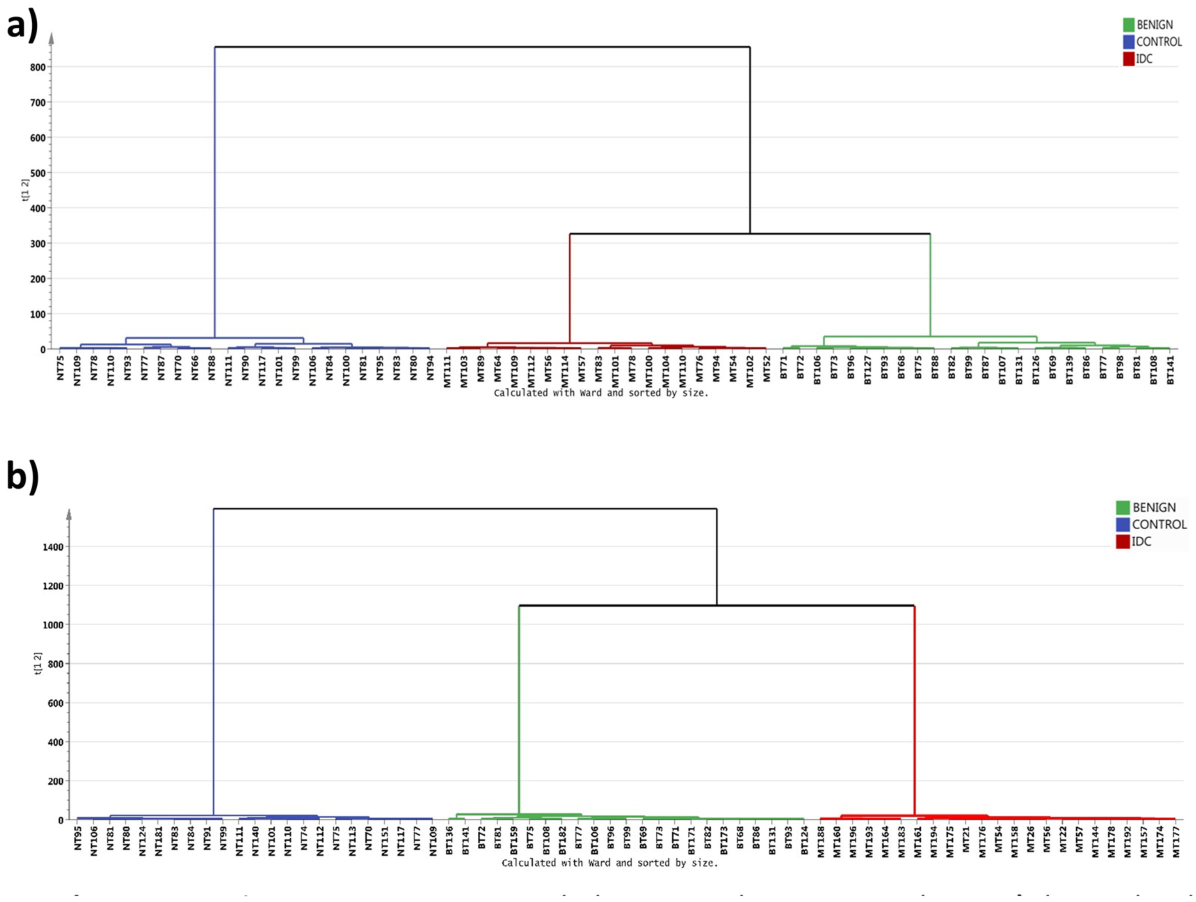

Supplementary Figure 3: Tissue metabolomics multivariate analysis. (a) hierarchical clustering analysis of tissue LC-MRM/MS data showing clustering of malignant (red), benign (green) and controls (blue), **(b)** hierarchical clustering analysis of tissue GC-MS data showing clustering of malignant (red), benign (green) and Control (blue).

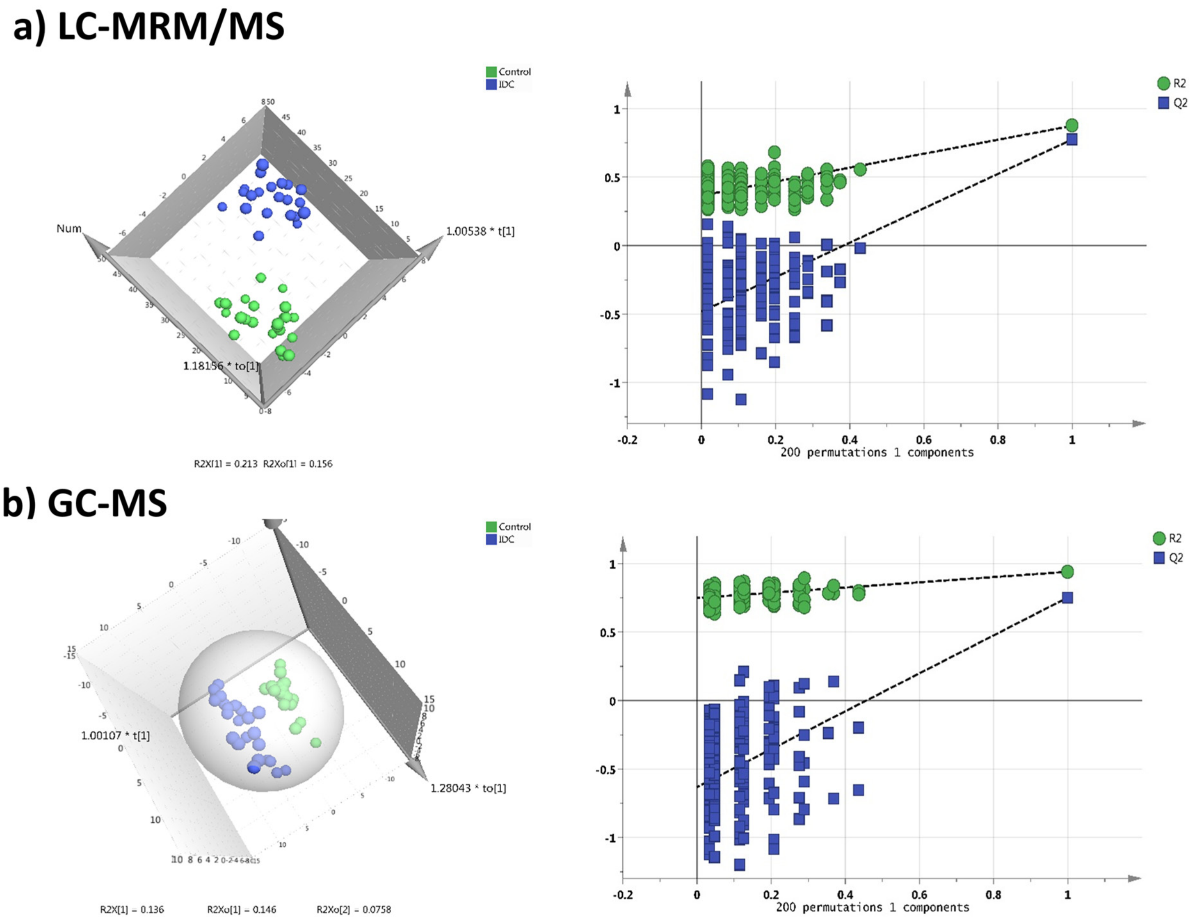

Supplementary Figure 4: Multivariate analysis of IDC Tissue against control (Normal tissue) **(a)** LC-MRM/MS OPLS-DA score plot along with permutation test plot, **(b)** GC-MS OPLS-DA score plot along with permutation test plot.

a) LC-MRM/MS

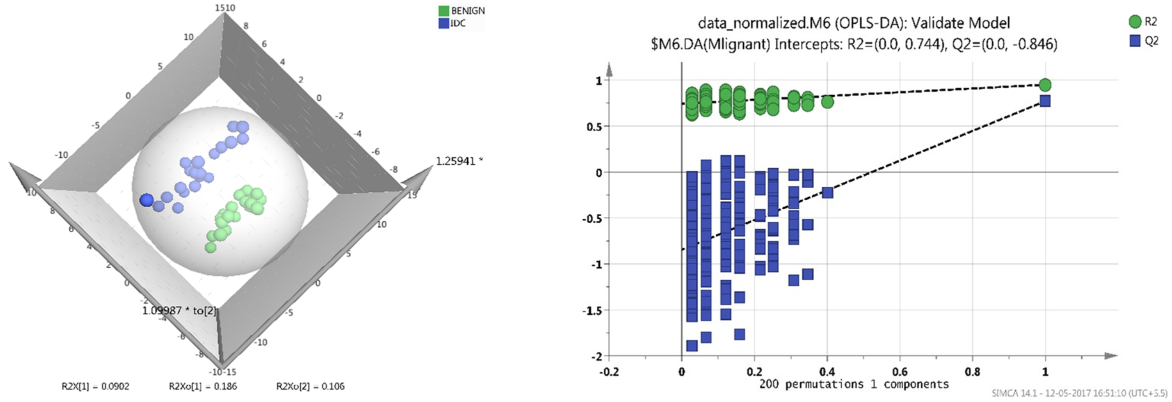

b) GC-MS

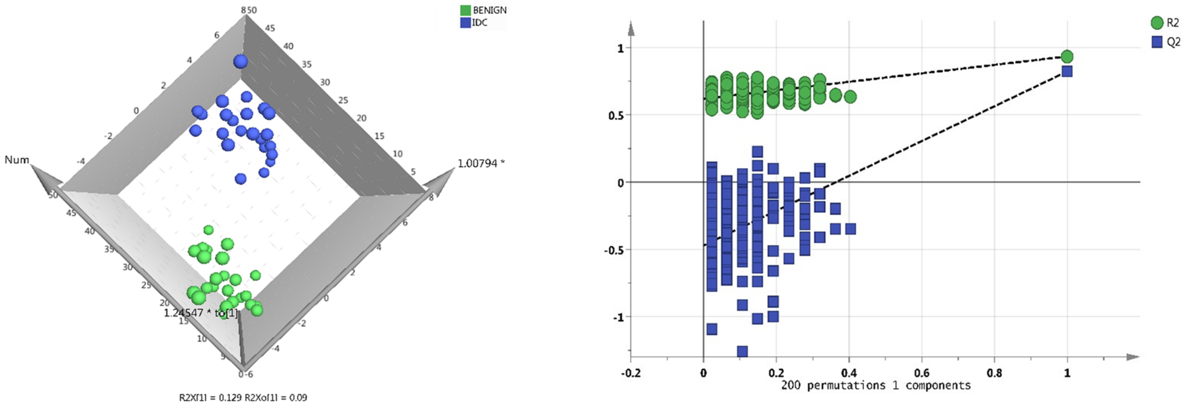

Supplementary Figure 5: Multivariate analysis of IDC tissue against benign **(a)** LC-MRM/MS OPLS-DA score plot along with permutation test plot, **(b)** GC-MS OPLS-DA score plot along with permutation test plot.

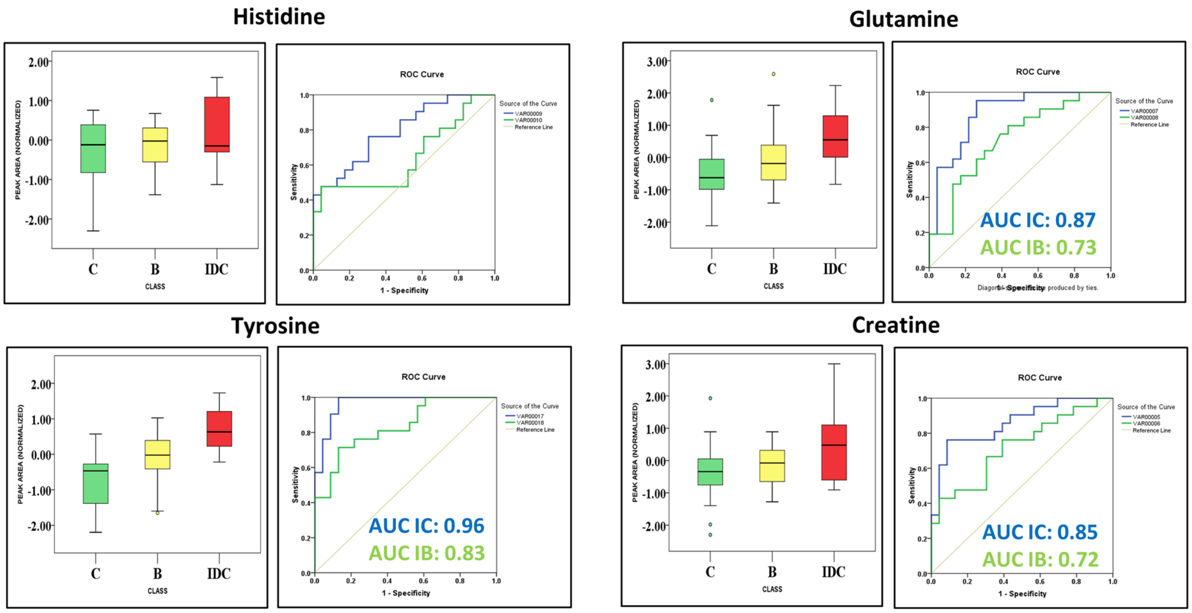

Supplementary Figure 6: Top four tissue metabolites discriminating IDC samples from benign samples and healthy controls. Box-and-whisker plots illustrating normalized concentration differences between control (C-Green box), Benign (B-Yellow box) and Invasive ductal carcinoma (IDC-Red box) along with ROC curve analysis plot of sensitivity versus specificity for the four metabolites predicting IDC samples against control (IC-blue line) and IDC samples against benign samples (IB-green line). The plot depicts higher discriminative ability of these metabolites for IDC samples than benign samples.

a)

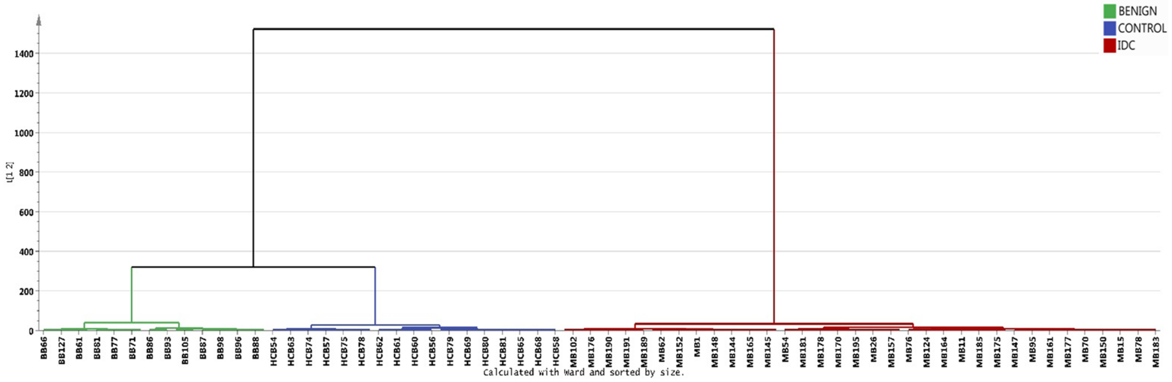

b)

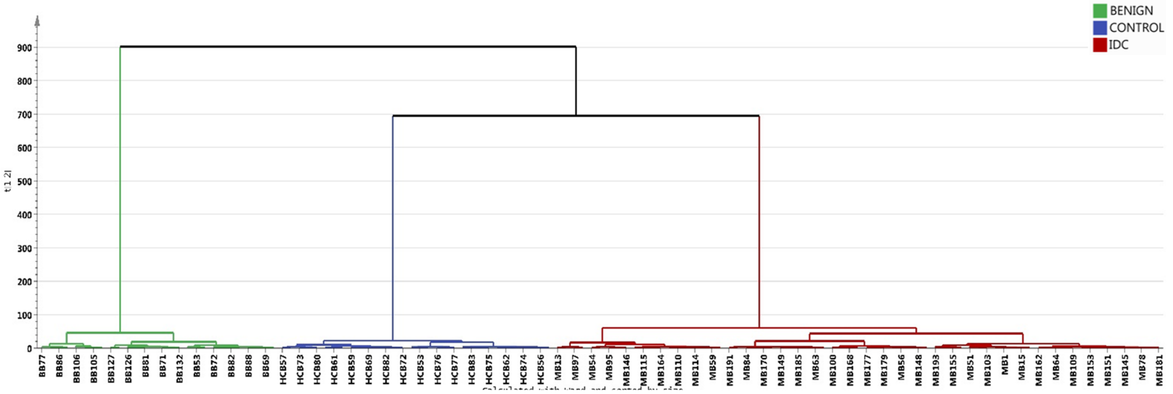

Supplementary Figure 7: Serum metabolomics multivariate analysis. (a) Hierarchical clustering analysis of serum LC-MRM/MS data showing clustering of IDC (red), benign (green) and controls (blue), **(b)** hierarchical clustering analysis of serum GC-MS data showing clustering of IDC (red), benign (green) and Control (blue).

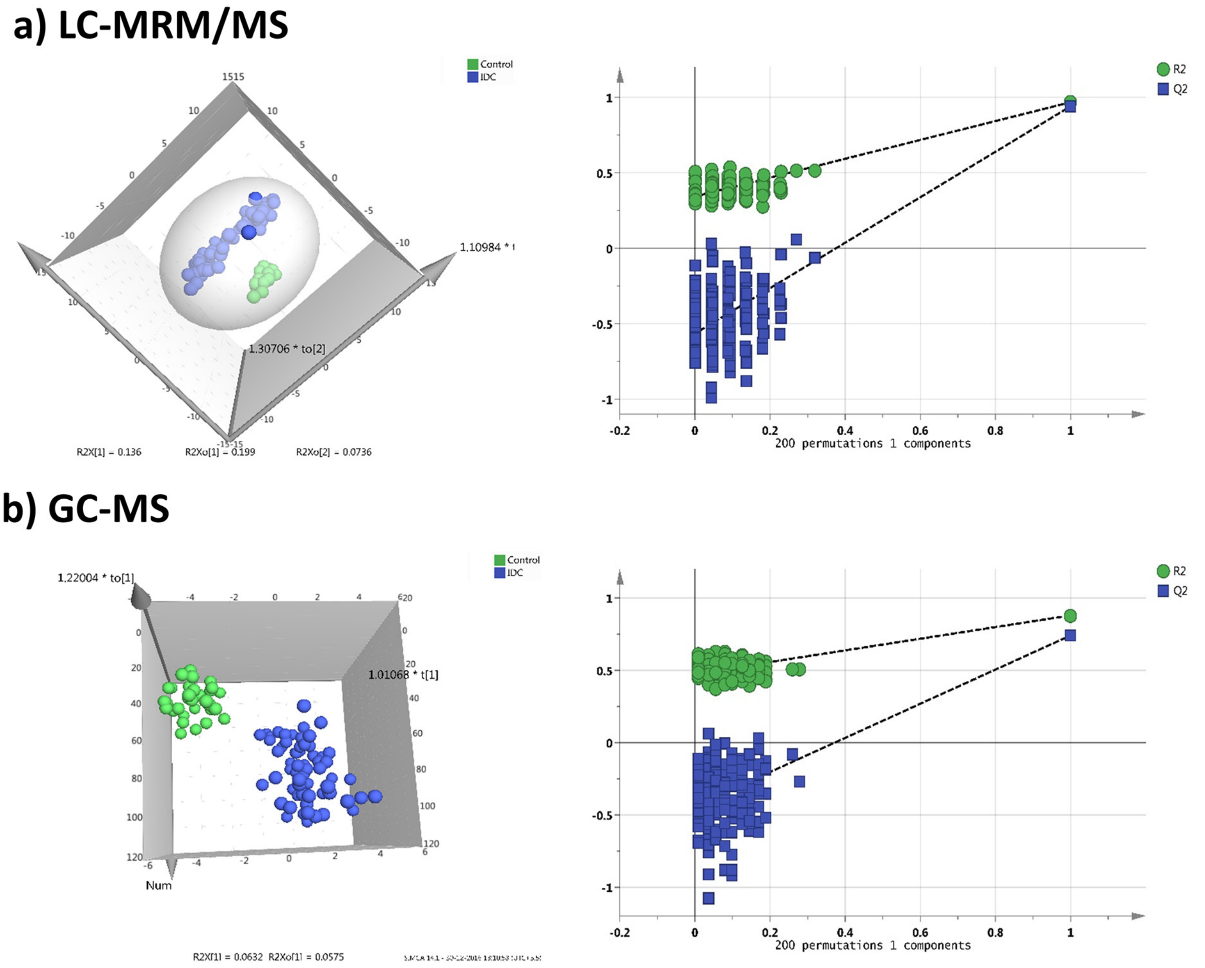

Supplementary Figure 8: Multivariate analysis of IDC serum against control **(a)** LC-MRM/MS OPLS-DA score plot along with permutation test plot, **(b)** GC-MS OPLS-DA score plot along with permutation test plot.

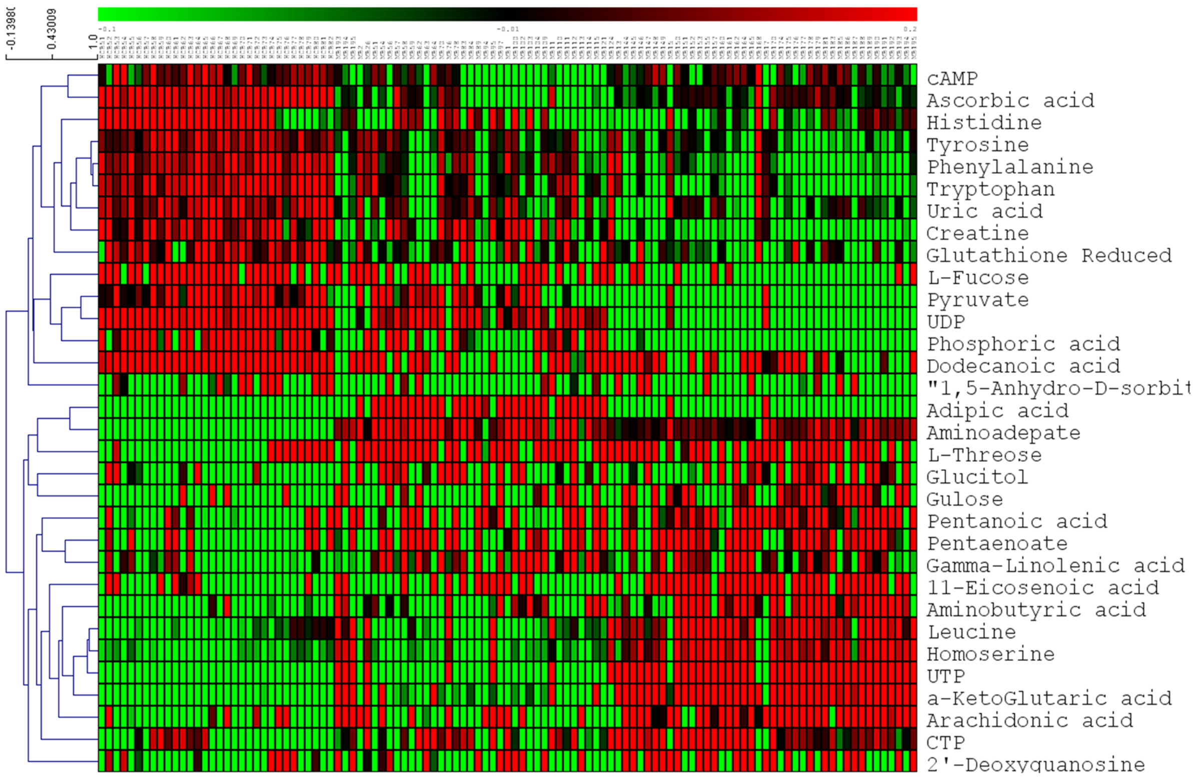

Supplementary Figure 9: Heatmap of 32 differential metabolites between IDC and controls in serum. The colours from green to red indicate the elevated amount of metabolites. (Control: Healthy control serum, IDC: Invasive ductal carcinoma serum).

a) LC-MRM/MS

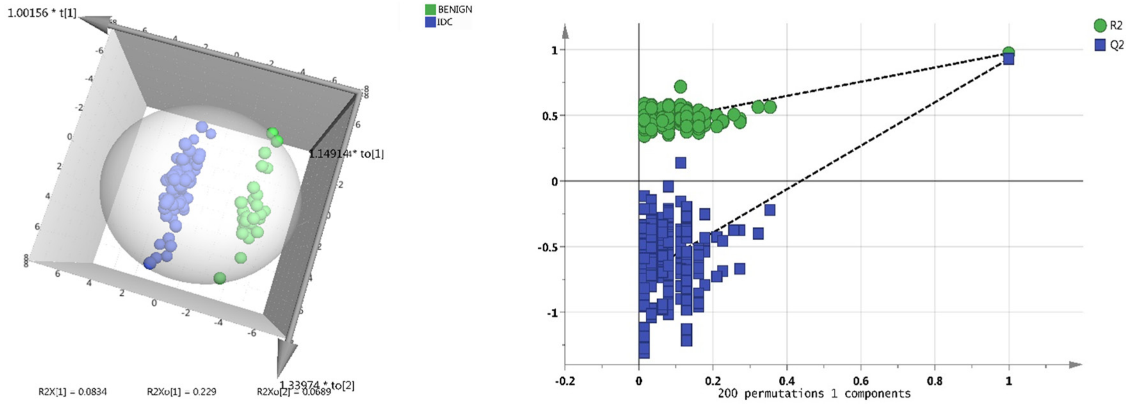

b) GC-MS

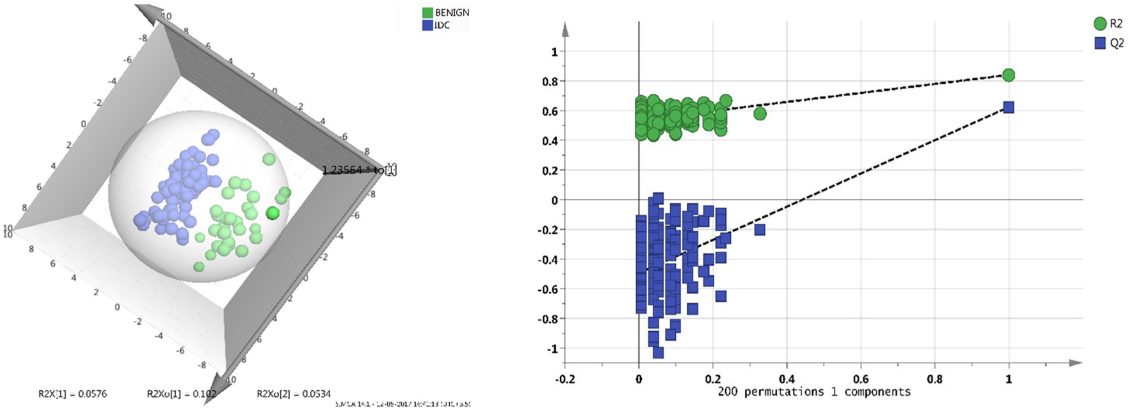

Supplementary Figure 10: Multivariate analysis of IDC serum against benign **(a)** LC-MRM/MS OPLS-DA score plot along with permutation test plot, **(b)** GC-MS OPLS-DA score plot along with permutation test plot.

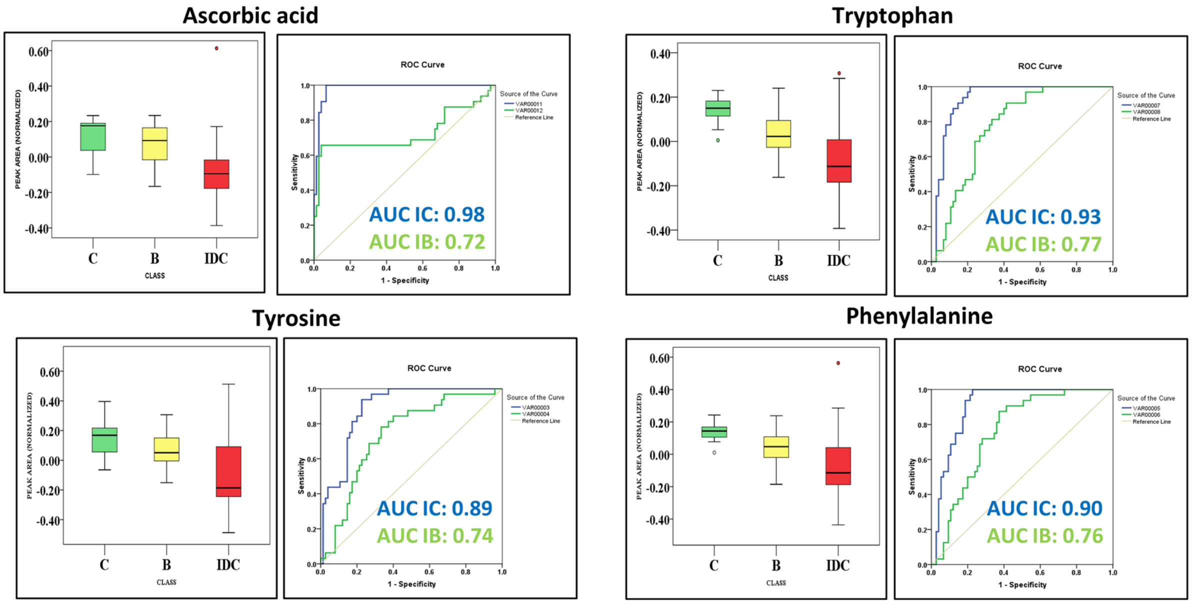

Supplementary Figure 11: Top four serum metabolites discriminating IDC samples from benign samples and healthy controls. Box-and-whisker plots illustrating normalized concentration differences between control (C-Green), Benign (B-Yellow) and Invasive ductal carcinoma (IDC-Red) along with ROC curve analysis plot of sensitivity versus specificity for the four metabolites predicting IDC samples against control (IC-blue line) and IDC samples against benign samples (IB-green line). The plot depicts higher discriminative ability of these metabolites for IDC samples than benign samples.

Supplementary Table 1: Tissue metabolites differentiating IDC from benign subjects a) Differential metabolites from LC-MRM/MS, b) Differential metabolites from GC-MS

See Supplementary File 1

Supplementary Table 2: Serum metabolites differentiating IDC from control subjects a) Differential metabolites from LC-MRM/MS, b) Differential metabolites from GC-MS

See Supplementary File 2

Supplementary Table 3: Serum metabolites differentiating IDC from benign subjects a) Differential metabolites from LC-MRM/MS, b) Differential metabolites from GC-MS

See Supplementary File 3

Supplementary Table 4: Metabolic pathways enriched in tissue

See Supplementary File 4
