# Supplementary material for: Metabolomic alterations in invasive ductal carcinoma of breast: A comprehensive metabolomic study using tissue and serum samples
Source: Oncotarget. 2017 Dec 23;9(2):2678–96. doi: 10.18632/oncotarget.23626 (PMC5788669; doi:10.18632/oncotarget.23626)
Supplement: Supplementary file 2 [file oncotarget-09-2678-s002.docx]

**Supplementary Table S1** Tissue metabolites differentiating IDC from benign subjects **a)** Differential metabolites from LC-MRM/MS, **b)** Differential metabolites from GC-MS.

| **S. No.** | **Metabolite** | **HMDB ID** | **VIP** | **p-value** | **FDR** | **FC** | **AUC** |
| --- | --- | --- | --- | --- | --- | --- | --- |
| **a) LC-MRM/MS** |  |  |  |  |  |  |  |
| 1 | Succinic acid | HMDB00254 | 2.24 | <0.01 | <0.01 | 0.05 | 0.98 |
| 2 | Methyl malonate | HMDB00202 | 2.19 | <0.01 | <0.01 | 0.05 | 0.98 |
| 3 | Uric acid | HMDB00289 | 1.72 | <0.01 | <0.01 | 0.00 | 0.97 |
| 4 | Hippuric acid | HMDB00714 | 1.96 | <0.01 | <0.01 | 0.02 | 0.94 |
| 5 | Tyrosine | HMDB00158 | 1.71 | <0.01 | <0.01 | 3.19 | 0.83 |
| 6 | Phenylalanine | HMDB00159 | 1.62 | <0.01 | <0.01 | 2.45 | 0.81 |
| 7 | a-KetoGlutaric acid | HMDB00208 | 1.50 | <0.01 | <0.01 | 0.23 | 0.81 |
| 8 | N-Acetylglycine | HMDB00532 | 1.39 | <0.01 | 0.02 | 2.63 | 0.79 |
| 9 | Lactic acid | HMDB00190 | 1.53 | <0.01 | 0.01 | 2.54 | 0.76 |
| 10 | Arabinose | HMDB29942 | 1.28 | <0.01 | 0.04 | 1.72 | 0.75 |
| 11 | Glutamine | HMDB00641 | 1.43 | 0.01 | 0.04 | 2.38 | 0.73 |
| 12 | Creatine | HMDB00064 | 1.45 | <0.01 | 0.04 | 2.36 | 0.72 |
| 13 | Leucine | HMDB00687 | 1.33 | <0.01 | 0.04 | 1.50 | 0.72 |
| 14 | Xylitol | HMDB02917 | 1.37 | 0.01 | 0.07 | 1.67 | 0.72 |
| 15 | Adonitol | HMDB00508 | 1.30 | 0.02 | 0.08 | 1.64 | 0.70 |
| 16 | Mono-Ethylmalonate | HMDB03282 | 1.26 | 0.01 | 0.08 | 1.41 | 0.70 |
| 17 | Methionine | HMDB00696 | 1.24 | 0.02 | 0.10 | 1.39 | 0.66 |
| 18 | Histidine | HMDB00177 | 1.30 | 0.01 | 0.06 | 2.29 | 0.64 |
| **b) GC-MS** |  |  |  |  |  |  |  |
| 19 | Stearic acid | HMDB00827 | 1.89 | <0.01 | <0.01 | 5.31 | 0.99 |
| 20 | Phosphoric acid | HMDB02142 | 1.83 | <0.01 | <0.01 | 4.44 | 0.89 |
| 21 | 2-Mannobiose | HMDB05775 | 1.75 | <0.01 | <0.01 | 3.38 | 0.89 |
| 22 | L-Aspartic acid | HMDB00191 | 1.38 | <0.01 | <0.01 | 0.36 | 0.83 |
| 23 | D-Allose | HMDB01151 | 1.32 | <0.01 | <0.01 | 0.23 | 0.82 |
| 24 | Pyrimidine | HMDB03361 | 1.43 | <0.01 | <0.01 | 0.29 | 0.82 |
| 25 | 9H-Purine | HMDB01366 | 1.58 | <0.01 | <0.01 | 0.32 | 0.81 |
| 26 | Cis-9-Hexadecenoic acid | HMDB03229 | 1.35 | <0.01 | <0.01 | 2.99 | 0.78 |
| 27 | Arachidonic acid | HMDB01043 | 1.42 | <0.01 | <0.01 | 5.50 | 0.78 |
| 28 | 11-Eicosenoic acid | HMDB34296 | 1.25 | <0.01 | 0.01 | 1.52 | 0.78 |
| 29 | L-Cysteine | HMDB00574 | 1.42 | <0.01 | <0.01 | 0.25 | 0.78 |
| 30 | Tocopherol | HMDB01492 | 1.24 | <0.01 | <0.01 | 5.54 | 0.78 |
| 31 | 4,7,10,13,16,19-Docosahexaenoic acid | HMDB02183 | 1.32 | <0.01 | <0.01 | 3.28 | 0.77 |
| 32 | Acetamide | HMDB31645 | 1.20 | <0.01 | 0.01 | 0.38 | 0.77 |
| 33 | Pentaenoate | HMDB00290 | 1.32 | <0.01 | 0.01 | 2.36 | 0.76 |
| 34 | 3,7-Cholest-5-ene | HMDB60441 | 1.29 | <0.01 | 0.01 | 0.35 | 0.76 |
| 35 | cis-7,10,13,16-Docosatetraenoic acid | HMDB07077 | 1.30 | <0.01 | 0.01 | 4.60 | 0.75 |
| 36 | 2,4-Pyrimidinedione | HMDB00300 | 1.23 | <0.01 | <0.01 | 6.04 | 0.75 |
| 37 | Decanedioic acid | HMDB00792 | 1.41 | <0.01 | <0.01 | 14.39 | 0.69 |

[Legends - HMDB ID: Metabolite ID obtained from HMDB database, VIP score: variable of importance score obtained from OPLS-DA plot (VIP>1.2), p value: p values obtained after performing t-test (p-value<0.05), FDR: value obtained after performing false discovery test, FC: fold change (FC>1.4), AUC: area under the curve value].
