# Supplementary material for: Metabolomic alterations in invasive ductal carcinoma of breast: A comprehensive metabolomic study using tissue and serum samples
Source: Oncotarget. 2017 Dec 23;9(2):2678–96. doi: 10.18632/oncotarget.23626 (PMC5788669; doi:10.18632/oncotarget.23626)
Supplement: Supplementary file 3 [file oncotarget-09-2678-s003.docx]

**Supplementary Table S2:** Serum metabolites differentiating IDC from control subjects **a)** Differential metabolites from LC-MRM/MS, **b)** Differential metabolites from GC-MS

| **S. No.** | **Metabolite** | **HMDB ID** | **VIP** | **p-value** | **FDR** | **FC** | **AUC** |
| --- | --- | --- | --- | --- | --- | --- | --- |
| **a) LC-MRM/MS** |  |  |  |  |  |  |  |
| 1 | Aminoadepate | HMDB00510 | 2.05 | <0.01 | <0.01 | 19.16 | 0.99 |
| 2 | Ascorbic acid | HMDB00044 | 1.73 | <0.01 | <0.01 | 0.31 | 0.98 |
| 3 | Tryptophan | HMDB00929 | 1.69 | <0.01 | <0.01 | 0.49 | 0.93 |
| 4 | Phenylalanine | HMDB00159 | 1.60 | <0.01 | <0.01 | 0.57 | 0.91 |
| 5 | UDP | HMDB00295 | 1.51 | <0.01 | <0.01 | 0.43 | 0.91 |
| 6 | Tyrosine | HMDB00158 | 1.53 | <0.01 | <0.01 | 0.61 | 0.89 |
| 7 | Uric acid | HMDB00289 | 1.39 | <0.01 | <0.01 | 0.60 | 0.88 |
| 8 | Pyruvate | HMDB00243 | 1.40 | <0.01 | <0.01 | 0.31 | 0.85 |
| 9 | Histidine | HMDB00177 | 1.42 | <0.01 | <0.01 | 0.50 | 0.84 |
| 10 | CTP | HMDB00082 | 1.39 | <0.01 | <0.01 | 3.72 | 0.83 |
| 11 | Creatine | HMDB00064 | 1.29 | <0.01 | <0.01 | 0.53 | 0.83 |
| 12 | a-KetoGlutaric acid | HMDB00208 | 1.34 | <0.01 | <0.01 | 15.26 | 0.81 |
| 13 | Glutathione Reduced | HMDB00125 | 1.22 | <0.01 | <0.01 | 0.48 | 0.81 |
| 14 | cAMP | HMDB00058 | 1.36 | <0.01 | <0.01 | 0.21 | 0.81 |
| 15 | Aminobutyric acid | HMDB00112 | 1.20 | <0.01 | <0.01 | 3.57 | 0.74 |
| 16 | Homoserine | HMDB00719 | 1.32 | <0.01 | <0.01 | 2.52 | 0.73 |
| 17 | Leucine | HMDB00687 | 1.22 | <0.01 | <0.01 | 1.62 | 0.71 |
| 18 | UTP | HMDB00285 | 1.42 | <0.01 | <0.01 | 52.09 | 0.67 |
| 19 | Adipic acid | HMDB00448 | 1.25 | <0.01 | <0.01 | 58.34 | 0.66 |
| **b) GC-MS** |  |  |  |  |  |  |  |
| 20 | Pentaenoate | HMDB00290 | 2.26 | <0.01 | <0.01 | 1.63 | 0.82 |
| 21 | Phosphoric acid | HMDB02142 | 1.87 | <0.01 | <0.01 | 0.46 | 0.85 |
| 22 | Pentanoic acid | HMDB00892 | 1.83 | <0.01 | <0.01 | 2.78 | 0.74 |
| 23 | Gulose | HMDB12326 | 1.72 | 0.01 | 0.05 | 2.03 | 0.76 |
| 24 | Arachidonic acid | HMDB01043 | 1.64 | <0.01 | <0.01 | 2.10 | 0.77 |
| 25 | Gamma-Linolenic acid | HMDB03073 | 1.58 | 0.02 | 0.08 | 1.72 | 0.73 |
| 26 | Dodecanoic acid | HMDB00638 | 1.58 | <0.01 | <0.01 | 0.71 | 0.70 |
| 27 | L-Fucose | HMDB00174 | 1.57 | <0.01 | <0.01 | 0.52 | 0.72 |
| 28 | L-Threose | HMDB02649 | 1.36 | <0.01 | <0.01 | 1.66 | 0.66 |
| 29 | 1,5-Anhydro-D-sorbitol | HMDB02712 | 1.34 | 0.01 | 0.05 | 0.41 | 0.58 |
| 30 | Glucitol | HMDB00247 | 1.30 | 0.01 | 0.05 | 2.85 | 0.69 |
| 31 | 11-Eicosenoic acid | HMDB34296 | 1.30 | <0.01 | 0.02 | 2.53 | 0.65 |
| 32 | 2'-Deoxyguanosine | HMDB00085 | 1.21 | 0.01 | 0.04 | 1.43 | 0.67 |

[Legends - HMDB ID: Metabolite ID obtained from HMDB database, VIP score: variable of importance score obtained from OPLS-DA plot (VIP>1.2), p value: p values obtained after performing t-test (p-value<0.05), FDR: value obtained after performing false discovery test, FC: fold change (FC>1.4), AUC: area under the curve value].
