# Supplementary material for: Metabolomic alterations in invasive ductal carcinoma of breast: A comprehensive metabolomic study using tissue and serum samples
Source: Oncotarget. 2017 Dec 23;9(2):2678–96. doi: 10.18632/oncotarget.23626 (PMC5788669; doi:10.18632/oncotarget.23626)
Supplement: Supplementary file 4 [file oncotarget-09-2678-s004.docx]

**Supplementary Table S3:** Serum metabolites differentiating IDC from benign subjects **a)** Differential metabolites from LC-MRM/MS, **b)** Differential metabolites from GC-MS

| **S. No.** | **Metabolite** | **HMDB ID** | **VIP** | **p-value** | **FDR** | **FC** | **AUC** |
| --- | --- | --- | --- | --- | --- | --- | --- |
| **a) LC-MRM/MS** |  |  |  |  |  |  |  |
| 1 | Aminoadepate | HMDB00510 | 2.26 | <0.01 | <0.01 | 17.01 | 1.00 |
| 2 | Cittruline | HMDB00904 | 1.28 | <0.01 | <0.01 | 8.01 | 0.83 |
| 3 | UDP | HMDB00295 | 1.58 | <0.01 | <0.01 | 0.46 | 0.83 |
| 4 | Histidine | HMDB00177 | 1.58 | <0.01 | <0.01 | 0.47 | 0.82 |
| 5 | Homoserine | HMDB00719 | 1.47 | <0.01 | <0.01 | 1.98 | 0.80 |
| 6 | Allantoin | HMDB00462 | 1.40 | <0.01 | <0.01 | 6.89 | 0.79 |
| 7 | UTP | HMDB00285 | 1.63 | <0.01 | <0.01 | 41.32 | 0.79 |
| 8 | Uric acid | HMDB00289 | 1.44 | <0.01 | <0.01 | 0.63 | 0.79 |
| 9 | Pyruvate | HMDB00243 | 1.39 | <0.01 | <0.01 | 0.40 | 0.78 |
| 10 | Tryptophan | HMDB00929 | 1.38 | <0.01 | <0.01 | 0.62 | 0.78 |
| 11 | Leucine | HMDB00687 | 1.33 | <0.01 | <0.01 | 1.49 | 0.77 |
| 12 | Phenylalanine | HMDB00159 | 1.41 | <0.01 | <0.01 | 0.66 | 0.76 |
| 13 | Creatine | HMDB00064 | 1.30 | <0.01 | <0.01 | 0.63 | 0.76 |
| 14 | a-KetoGlutaric acid | HMDB00208 | 1.29 | <0.01 | <0.01 | 5.14 | 0.74 |
| 15 | Tyrosine | HMDB00158 | 1.29 | <0.01 | <0.01 | 0.68 | 0.74 |
| 16 | Ascorbic acid | HMDB00044 | 1.44 | <0.01 | <0.01 | 0.32 | 0.73 |
| 17 | Adipic acid | HMDB00448 | 1.37 | <0.01 | <0.01 | 65.05 | 0.54 |
| **b) GC-MS** |  |  |  |  |  |  |  |
| 18 | DL-Ornithine | HMDB32455 | 1.78 | <0.01 | 0.01 | 1.41 | 0.83 |
| 19 | 5-Uridine | HMDB00296 | 1.63 | <0.01 | <0.01 | 0.41 | 0.81 |
| 20 | L-Threitol | HMDB02994 | 1.24 | 0.00 | 0.02 | 0.57 | 0.79 |
| 21 | Undecanoic acid | HMDB00947 | 1.27 | 0.01 | 0.06 | 0.20 | 0.79 |
| 22 | Phosphoric acid | HMDB02142 | 1.88 | <0.01 | <0.01 | 0.21 | 0.78 |
| 23 | 8,11,14-Eicosatrienoic acid | HMDB02925 | 1.61 | <0.01 | <0.01 | 0.13 | 0.77 |
| 24 | Butanoic acid | HMDB00039 | 1.83 | <0.01 | <0.01 | 0.69 | 0.76 |
| 25 | Tocopherol | HMDB01492 | 1.67 | <0.01 | <0.01 | 1.61 | 0.74 |
| 26 | 3,4-Dihydroxybutanoic acid | HMDB00337 | 1.55 | <0.01 | <0.01 | 0.59 | 0.73 |
| 27 | Stearic acid | HMDB00827 | 1.90 | <0.01 | <0.01 | 1.73 | 0.73 |
| 28 | n-Acetyl-L-Lysine | HMDB01550 | 1.78 | <0.01 | 0.01 | 1.41 | 0.72 |
| 29 | Heptanedioic acid | HMDB00857 | 1.30 | <0.01 | 0.01 | 1.58 | 0.69 |
| 30 | Hexanoic acid | HMDB00535 | 1.21 | 0.01 | 0.03 | 0.43 | 0.68 |
| 31 | Eicosanoic acid | HMDB02212 | 1.30 | 0.02 | 0.06 | 0.30 | 0.65 |
| 32 | 3-Isoxazolidinone | HMDB39426 | 1.28 | <0.01 | 0.01 | 0.50 | 0.64 |
| 33 | Thymine | HMDB00262 | 1.64 | <0.01 | <0.01 | 1.90 | 0.61 |
| 34 | 9-Octadecenoic acid | HMDB00207 | 1.45 | <0.01 | <0.01 | 0.44 | 0.58 |
| 35 | 2-Keto valeric acid | HMDB01865 | 1.34 | <0.01 | <0.01 | 0.61 | 0.53 |

[Legends - HMDB ID: Metabolite ID obtained from HMDB database, VIP score: variable of importance score obtained from OPLS-DA plot (VIP>1.2), p value: p values obtained after performing t-test (p-value<0.05), FDR: value obtained after performing false discovery test, FC: fold change (FC>1.4), AUC: area under the curve value].
