# Supplementary material for: Metabolomic alterations in invasive ductal carcinoma of breast: A comprehensive metabolomic study using tissue and serum samples
Source: Oncotarget. 2017 Dec 23;9(2):2678–96. doi: 10.18632/oncotarget.23626 (PMC5788669; doi:10.18632/oncotarget.23626)
Supplement: Supplementary file 5 [file oncotarget-09-2678-s005.docx]

**Supplementary Table S4:** Metabolic pathways enriched in tissue.

| **Sr. No.** | **Pathway** | **Total** | **Hits** | **Raw p** | **FDR** |
| --- | --- | --- | --- | --- | --- |
| 1 | Nitrogen metabolism | 39 | 7 | <0.01 | <0.01 |
| 2 | Pyrimidine metabolism | 60 | 6 | <0.01 | 0.01 |
| 3 | Aminoacyl-tRNA biosynthesis | 75 | 5 | 0.01 | 0.12 |
| 4 | Fatty acid biosynthesis | 49 | 4 | 0.01 | 0.12 |
| 5 | D-Glutamine and D-glutamate metabolism | 11 | 2 | 0.01 | 0.19 |
| 6 | Riboflavin metabolism | 21 | 2 | 0.04 | 0.51 |
| 7 | Purine metabolism | 92 | 4 | 0.05 | 0.51 |
| 8 | Alanine, aspartate and glutamate metabolism | 24 | 2 | 0.05 | 0.51 |
| 9 | Phenylalanine, tyrosine and tryptophan biosynthesis | 27 | 2 | 0.06 | 0.54 |
| 10 | beta-Alanine metabolism | 28 | 2 | 0.07 | 0.54 |
| 11 | Arginine and proline metabolism | 77 | 3 | 0.11 | 0.76 |
| 12 | Glutathione metabolism | 38 | 2 | 0.11 | 0.76 |
| 13 | Histidine metabolism | 44 | 2 | 0.15 | 0.81 |
| 14 | Ascorbate and aldarate metabolism | 45 | 2 | 0.15 | 0.81 |
| 15 | Phenylalanine metabolism | 45 | 2 | 0.15 | 0.81 |
| 16 | Taurine and hypotaurine metabolism | 20 | 1 | 0.27 | 1.00 |
| 17 | Thiamine metabolism | 24 | 1 | 0.31 | 1.00 |
| 18 | Fatty acid elongation in mitochondria | 27 | 1 | 0.34 | 1.00 |
| 19 | Pantothenate and CoA biosynthesis | 27 | 1 | 0.34 | 1.00 |
| 20 | Glycolysis or Gluconeogenesis | 31 | 1 | 0.38 | 1.00 |
| 21 | Pyruvate metabolism | 32 | 1 | 0.39 | 1.00 |
| 22 | Propanoate metabolism | 35 | 1 | 0.42 | 1.00 |
| 23 | Ubiquinone and other terpenoid-quinone biosynthesis | 36 | 1 | 0.43 | 1.00 |
| 24 | Inositol phosphate metabolism | 39 | 1 | 0.46 | 1.00 |
| 25 | Butanoate metabolism | 40 | 1 | 0.46 | 1.00 |
| 26 | Galactose metabolism | 41 | 1 | 0.47 | 1.00 |
| 27 | Primary bile acid biosynthesis | 47 | 1 | 0.52 | 1.00 |
| 28 | Glycine, serine and threonine metabolism | 48 | 1 | 0.53 | 1.00 |
| 29 | Fatty acid metabolism | 50 | 1 | 0.54 | 1.00 |
| 30 | Pentose and glucuronate interconversions | 53 | 1 | 0.56 | 1.00 |
| 31 | Arachidonic acid metabolism | 62 | 1 | 0.62 | 1.00 |
| 32 | Tyrosine metabolism | 76 | 1 | 0.70 | 1.00 |
| 33 | Amino sugar and nucleotide sugar metabolism | 88 | 1 | 0.75 | 1.00 |
| 34 | Porphyrin and chlorophyll metabolism | 104 | 1 | 0.81 | 1.00 |

[Legends - Total: total number of compounds in the pathway, Hit: actually matched number from the data, p value: p value calculated from the enrichment analysis, FDR: p value adjusted using False Discovery Rate]
